# Comparison of machine learning and logistic regression as predictive models for adverse maternal and neonatal outcomes of preeclampsia: A retrospective study

**DOI:** 10.3389/fcvm.2022.959649

**Published:** 2022-10-12

**Authors:** Dongying Zheng, Xinyu Hao, Muhanmmad Khan, Lixia Wang, Fan Li, Ning Xiang, Fuli Kang, Timo Hamalainen, Fengyu Cong, Kedong Song, Chong Qiao

**Affiliations:** ^1^State Key Laboratory of Fine Chemicals, Dalian R&D Center for Stem Cell and Tissue Engineering, Dalian University of Technology, Dalian, China; ^2^Department of Obstetrics and Gynecology, Second Affiliated Hospital of Dalian Medical University, Dalian, China; ^3^Faculty of Information Technology, University of Jyvaskyla, Jyväskylä, Finland; ^4^School of Biomedical Engineering, Faculty of Electronic Information and Electrical Engineering, Dalian University of Technology, Dalian, China; ^5^Institute of Zoology, University of Punjab, Lahore, Pakistan; ^6^Department of Obstetrics and Gynecology, Shengjing Hospital, China Medical University, Shenyang, China; ^7^Department of Obstetrics and Gynecology, Jingzhou Hospital Affiliated to Yangtze University, Jingzhou, China; ^8^School of Artificial Intelligence, Faculty of Electronic Information and Electrical Engineering, Dalian University of Technology, Dalian, China; ^9^Key Laboratory of Integrated Circuit and Biomedical Electronic System, Dalian University of Technology, Dalian, China

**Keywords:** pre-eclampsia (PE), adverse outcomes, maternal, neonatal, predictive models, machine learning, logistic regression, retrospective study

## Abstract

**Introduction:**

Preeclampsia, one of the leading causes of maternal and fetal morbidity and mortality, demands accurate predictive models for the lack of effective treatment. Predictive models based on machine learning algorithms demonstrate promising potential, while there is a controversial discussion about whether machine learning methods should be recommended preferably, compared to traditional statistical models.

**Methods:**

We employed both logistic regression and six machine learning methods as binary predictive models for a dataset containing 733 women diagnosed with preeclampsia. Participants were grouped by four different pregnancy outcomes. After the imputation of missing values, statistical description and comparison were conducted preliminarily to explore the characteristics of documented 73 variables. Sequentially, correlation analysis and feature selection were performed as preprocessing steps to filter contributing variables for developing models. The models were evaluated by multiple criteria.

**Results:**

We first figured out that the influential variables screened by preprocessing steps did not overlap with those determined by statistical differences. Secondly, the most accurate imputation method is K-Nearest Neighbor, and the imputation process did not affect the performance of the developed models much. Finally, the performance of models was investigated. The random forest classifier, multi-layer perceptron, and support vector machine demonstrated better discriminative power for prediction evaluated by the area under the receiver operating characteristic curve, while the decision tree classifier, random forest, and logistic regression yielded better calibration ability verified, as by the calibration curve.

**Conclusion:**

Machine learning algorithms can accomplish prediction modeling and demonstrate superior discrimination, while Logistic Regression can be calibrated well. Statistical analysis and machine learning are two scientific domains sharing similar themes. The predictive abilities of such developed models vary according to the characteristics of datasets, which still need larger sample sizes and more influential predictors to accumulate evidence.

## Introduction

Preeclampsia affects about 5 to 7% of all pregnant women but is responsible for over 70,000 maternal deaths and 500,000 fetal deaths worldwide every year (1). As a placenta-mediated disease, the pathogenesis of preeclampsia is poorly understood, and therapeutic interventions are limited. The only effective treatment is the termination of the pregnancy, which may cause severe consequences of maternal target-organ damage or neonatal concomitant prematurity (2). Therefore, both accurate prediction of the disease onset for expectant women and precise identification of susceptible patients for adverse maternal or neonatal outcomes are important for required intensive monitoring and preventive management.

Recently, early screening models with promising biomarkers effectively discriminate suspected women from normal pregnancies (3), while significant heterogeneity can still be revealed when these models are utilized to predict adverse maternal and perinatal outcomes (4). Considering the time span from pre-pregnancy or early pregnancy to the appearance of adverse outcomes, applying short-term predictive models may be more realistic in ruling out the occurrence of adverse events in women with preeclampsia. Furthermore, novel biomarkers that present exciting roles for assisting prediction are still under testing for extensive clinical utility. Therefore, deep mining the application of maternal demographic characteristics, medical history, physical examination, as well as biochemical indicators obtained during antenatal care visits, or even obtained just ahead of confronting pregnancy outcomes, and evaluating the reliability of real-time predictive models developed by these variables, will still be an efficient and economical choice for timely distinguishing adverse outcomes, especially in low-resource settings.

For the purpose of developing short-term predictive models with common clinical observations and laboratory tests, we derived the variables collected routinely before the termination of pregnancy based on revealing the actual individual signaling when they confront pregnancy outcomes. The developed predictive models with variables can be considered bottom-up work that laid a foundation for subsequent real-time predictive models embedded in different trimesters of pregnancy.

The traditional predictive model assisting clinical decision-making for binary outcomes is logistic regression (LR). This kind of regression-type modeling is mainly based on assumptions and probability calculations, and the interpretation of models specified by artificial intervention and background knowledge is quite essential (5). Nowadays, “machine learning” (ML) has become a prevalent approach for modeling, encompassing a variety of algorithmic strategies (6). It is claimed that ML models learn from data directly and automatically with highly flexible algorithms. With increasing computational power, the capability of ML-based predictive models has vastly expanded, providing an opportunity for accurate prediction from voluminous electronic medical records (7). As a matter of fact, the distinction between LR and ML is blurry. “Continuum” may be a more appropriate phrase to describe their relationship (8). Though the algorithmic flexibility promises ML to perform better over traditional statistical modeling on handling a large number of data, which may be especially preferred by preeclampsia, with heterogeneous pathophysiology and clinical presentation (9), the fair comparison between ML and LR is still sparsely exploited. In other words, the reliability of different models performed on a certain type of data or a certain sample size still needs assessment. To date, limited research studies focused on the development of ML models for preeclampsia predictive tasks, which mainly involved early detection of preeclampsia (10–14), exploring identified biomarkers (15), or assessment of subsequent cardiovascular risk (16), while there is still no research designed to predict adverse pregnancy outcomes.

Therefore, the primary objective of this study was to evaluate the performance of ML and LR for the development of short-term predictive models for binary maternal or neonatal outcomes involving pre-eclampsia, and the models were generated by common clinical indicator. The secondary objective was to further explore the characteristics of these models when applied to our structural medical recodes, aiming to provide information for the match-up patterns of the two modeling approaches, which are from two different domains but share similar themes.

## Methods

**Figure 1:**
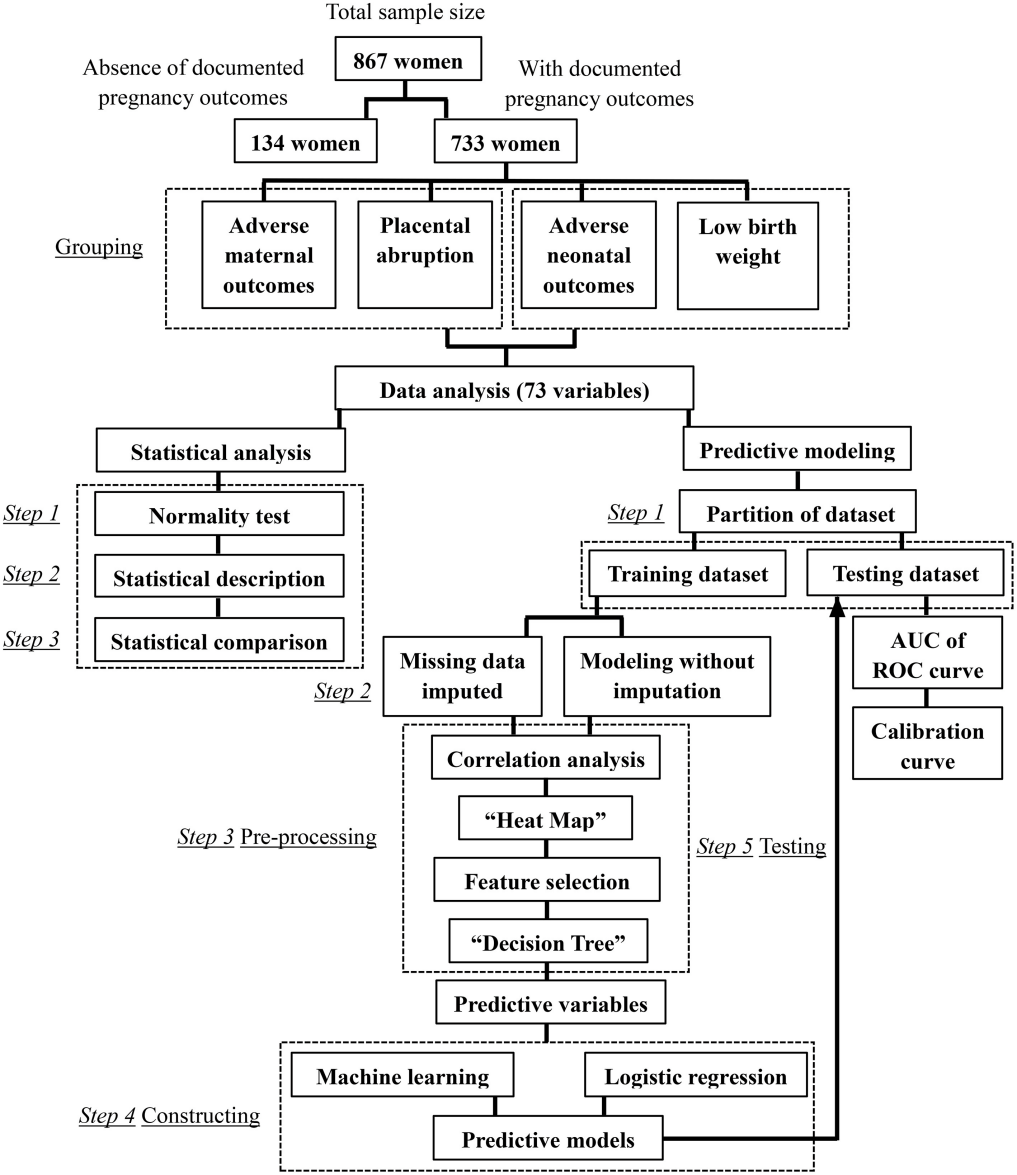
The flowchart of this study.

The flow chart of this research can be seen in Figure 1.

### Study population

A retrospective cohort study consisted of women admitted to two tertiary care hospitals with delivery services in China, Second Affiliated Hospital of Dalian Medical University and Shengjing Hospital of China Medical University, from January 1, 2007, to December 31, 2017. Medical records were reviewed, and 867 Han-Chinese women diagnosed with preeclampsia were enrolled. Seven hundred thirty-three pregnant women had definite records of pregnancy outcomes, while 134 cases were admitted to hospitals after the confirmation of the diagnosis of preeclampsia, and the pregnancy outcomes are unknown for planned discharge or required transfer. Individuals were excluded from the study when the missing data rates of analyzed variables were more than 60%.

The diagnostic criteria for preeclampsia were based on the ACOG (American College of Obstetricians and Gynecologists) practice bulletin (17).

The requirement for informed consent was waived off for this retrospective and observational study. Personal information of the subjects was shielded before any analysis. The study protocols were approved by the Ethics Committees of the two hospitals, and all procedures adhered to the ethical standards outlined in the principles of the Declaration of Helsinki.

### Grouping

Eight hundred sixty-seven participants were initially divided into the early-onset group (EOP, early-onset of preeclampsia would be labeled when preeclampsia presents before 34 weeks (2), *n* = 372) and the late-onset group (LOP, *n* = 495).

Then, the 733 women with well-documented pregnancy outcomes were categorized separately based on different adverse outcomes. (1) Grouped based on adverse or satisfactory maternal outcomes: the adverse maternal outcomes group (AMO, *n* = 182) and the control group (CON-AMO, *n* = 551); (2) grouped based on placental abruption occurred or not: as a more specific adverse maternal outcome, the differences between placental abruption group (PA, *n* = 71), and the control group (CON-PA, *n* = 662) were also identified; (3) grouped based on adverse or satisfactory neonatal outcomes: the adverse neonatal outcomes group (ANO, *n* = 423) and the control group (CON-ANO, *n* = 310); (4) grouped based on low birth weight occurred or not: the low birth weight group (LBW, *n* = 253) and the control group (CON-LBW, *n* = 480).

The adverse maternal and neonatal outcomes regarding preeclampsia were identified according to the international consensus (18).

Maternal adverse outcomes include maternal mortality, eclampsia, stroke, cortical blindness, retinal detachment, pulmonary edema, acute kidney injury, liver capsule hematoma or rupture, placental abruption, postpartum hemorrhage, raised liver enzymes, low platelets, admission to ICU required, intubation, and mechanical ventilation.

Outcomes demonstrating the impact of preeclampsia on the fetus and neonate include stillbirth, preterm, low birth weight, small-for-gestational-age, neonatal mortality, neonatal seizures, admission to neonatal ICU, and respiratory support.

### Collection of variables

All the maternal variables were obtained within 24 h after admission, and all the neonatal variables were recorded after delivery; all the biological samples were analyzed in the laboratories of two hospitals. The list of variables can be seen in Supplementary Table 1.

Gestational ages were confirmed by ultrasonic examinations before 14 gestational weeks.

Hypoalbuminemia is defined as plasmatic albumin < 30 g/L (19).

The diagnosis criteria of “impaired liver function,” “renal insufficiency,” “thrombocytopenia,” and “HELLP syndrome” were all defined by the ACOG practice bulletin (17).

The “creatinine clearance rates” were calculated based on the Cockcroft-Gault equation (20).

### Imputation for missing values

Random missing data were inevitable in our retrospective study to unnecessarily threaten the validation of results. Therefore, imputation techniques were proposed before any subsequent analysis.

To observe the influence of imputation on the datasets and select the appropriate imputed method, the four datasets were split into training (70%) and testing (30%) sub-datasets randomly, and the training datasets were either imputed or not. Meanwhile, several imputation techniques were applied when the training datasets required imputation, and the imputation accuracy was evaluated by training on the Random Forest (RF) classifier.

Both Iterative Imputer and K-Nearest Neighbor (KNN) (21) were proposed as imputed techniques with the necessary three-fold iteration. As Iterative Imputers, Bayesian Ridge Regression and Extratree were the selected algorithms, which impute each missing value several times until algorithmic convergence is reached in each model (22). The principle of KNN is that the value can be approximated by the “k” neighbors closest to it. Depending on the chosen values of “k,” the efficiency of imputation varied (22).

After data imputation, the missing data rate was calculated.

### Statistical description and analysis

Firstly, the normality of distribution was analyzed by the Shapiro–Wilk test for continuous variables. Secondly, intergroup comparisons between continuous variables with normal distributions were performed by Student's *t*-test and presented as mean ± standard deviation, while continuous variables with skewed distributions were compared using the Mann–Whitney *U*-test and described as median with interquartile range. Thirdly, categorical variables were analyzed by the Chi-square test or Fisher's exact test. Finally, ordinal variables were compared by the Mann-Whitney *U*-test. A probability level of *P*-value <0.05 was taken as statistically significant.

All analyses were performed by SPSS version 26 (IBM Corp., Armonk, NY, USA), Python language version 3.6.9, and GraphPad Prism 6.01 (GraphPad Software, San Diego, CA, United States).

### Selection of predictive variables

The selection of predictive variables correlated with clinical outcomes was performed by two different strategies sequentially: the first step is statistical correlation analysis, and the second step is feature selection performed by the decision tree (DT) algorithm.

Statistical correlation analysis is applied to calculate the association between two variables. Pearson correlation is typically used for jointly normally distributed continuous data, while Spearman rank correlation can be used for non-normally distributed data (23). The correlation coefficient shows the correlated value of changes, and the preceding sign indicates the direction of correlated changes.

The “heat maps” can be constructed according to the results of correlation analysis to solve the problems of pairwise graphic mapping of variables simultaneously and to assess the presence of dependence in an illustrative way. The independent variables screened by correlation analysis will be obtained for the following exploration.

Further feature selection was performed by DT models as another preprocessing measure for the following construction of predictive models (the explanation of this model will be discussed below in detail). The area under the receiver operating characteristic (ROC) curve, also known as AUC, is commonly used for ranking the performance of models (24). Each variable was applied to the DT model, and the performance of each model was evaluated by the AUC value. With AUC values >0.5, variables would be filtered as the targeted satisfactory predictors for the following predictive models (25).

### Selection of predictive models

All the selected variables were standardized to the same order of magnitude and normalized from zero to one. The standardization was essential to weaken or even eliminate the disturbance factors of variables with different features, thus solving the problems of comparability between different variables and improving the prediction accuracy (26).

The selection of predictive models for clinical outcome classification tasks was also performed by two different strategies separately, LR and ML algorithms. These models originate in two different communities—statistics and computer science—but share many similarities. After the variable selection scheme was used to remove spurious variables, LR (26) and six ML prediction models were developed from the split training datasets, and the validation of models was tested on the testing datasets. GridSearch with Cross-Validation was the applied parameter optimization technique (27). The discriminative power of the models was assessed by the AUC of the ROC curve, and the calibration quality was determined by the calibration curve.

**Figure 2:**
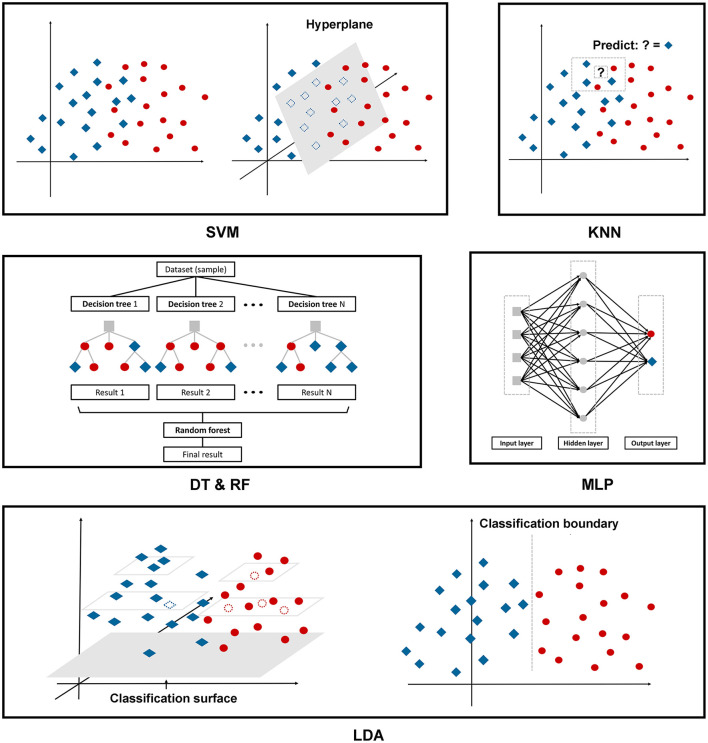
The illustration of the machine learning algorithms applied in this research.

The following are the six ML algorithms we applied to construct the models, and Figure 2 depicts these algorithms in a more illustrative way.

A support vector machine (SVM) is a binary linear classifier for classification or regression analysis. SVM can achieve reasonable accuracy from small data sets by creating a decision boundary between two classes and optimizing the distance of the hyperplane between the boundary points to separate the different classes, which enables the prediction of labels from one or more feature vectors (28).

KNN, as we mentioned above, is one of the oldest, simplest, and most accurate algorithms for pattern classification and regression models. This classifier depends mainly on measuring the distance or similarity between the tested and training examples. There is no fixed number of parameters, no data size limitation, and no data distribution assumptions (29).

The DT classifier is a single base classifier consisting of nodes and edges. Starting from the root node, also known as the first split point, the split determines the divisions of the entire dataset based on calculation. The process continues from top to bottom until no more partitioning is required, and the leaves present at the end of the decision tree represent the last partitions (30).

RF is an ensemble learning method to overcome the drawbacks of a single base prediction model, aiming to achieve higher accuracy even though the dataset size is very small. This model includes multiple decision trees corresponding to various sub-datasets created from an identical dataset. The model can be trained with a different subset of features rather than selecting the best feature present in the dataset (30).

The Multi-Layer Perceptron (MLP) is a feedforward artificial neural network with a high degree of connectivity determined by the synaptic weights of the network, consisting of input, hidden, and output layers. Employing the backpropagation algorithm, we fixed the synaptic weights as the signal propagated in the forward phase, while in the backward phase, the error signal propagates backward until it reaches the synaptic weight and is adjusted (31).

Linear discriminant analysis (LDA) is a multivariate classification technique. This model seeks a linear combination to discriminate multiple measures into two different groups. The decision boundary obtained from the testing sample plays a crucial role in the correct recognition. Data from a higher dimensional space is performed as a linear transformation to a lower dimensional space to achieve the final decision (32).

For all ML models, the ten-fold cross-validation technique was optimized to select the best bias-corrected discriminant model. Data are randomly divided into ten equal-sized sets, and in each iteration, seven sets are utilized for training, and three sets are utilized for testing. After ten iterations are performed, each set can be used as a testing set in a rotatory manner. The final performance of models is calculated as the average of all the iterations (33).

## Results

### Population characteristics

A total of 867 pregnant women were included in our study. Among them, there are 733 cases with documented pregnancy outcomes. Seventy-three variables were extracted from medical records, including demographics, pregnancy complications, features of deliveries and neonates, and maternal physical and laboratory examinations. The missing data rate for 73 variables is indicated in Supplementary Table 1.

The proportion of early-onset type is 42.9%, while the rates of adverse maternal outcomes, placental abruption, adverse neonatal outcomes, and low birth rate are 24.8, 9.7, 57.7, and 34.5%, separately. Up to 14.5% of women developed preeclampsia with superimposed chronic hypertension. Twenty-one point three percentage of cases were complicated by pre-gestational or gestational diabetes.

### Adverse maternal outcomes

Firstly, seven imputation strategies were proposed, including Bayesian Ridge Regression, Extratree, and KNN, when the value for the “k” nearest neighbor was equal to 2, 3, 5, 7, and 9. According to the results of the accuracy comparison (Supplementary Figure 1), it can be verified that the preferred imputation strategy was KNN imputer with nine neighbors overall, and all the datasets were imputed by this method consequently. The missing data rate can be seen in Supplementary Table 1.

Considering 733 women with well-documented pregnancy outcomes, we first grouped the participants according to adverse or satisfactory maternal outcomes. The statistical description and comparison between AMO and CON-AMO groups are shown in Supplementary Table 2, while variables with statistical significance are listed in Table 1.

**Table 1:** Variables with statistical significance between adverse maternal outcomes group and control group.

**Variables**		**Adverse maternal outcomes**	**Placental abruption**
		**AMO vs. CON-AMO**	**PA vs. CON-PA**
**Demography**			
Gravidity		2 (1–3) vs. 2 (1–3)*	2 (1–3) vs. 2 (1–3)*
**Complications**			
Early-onset type	Yes	117 (64.3%) vs. 159 (28.9%)***	49 (69.0%) vs. 227 (34.3%)***
Maternal hypoproteinemia	Yes	70 (38.5%) vs. 86 (15.6%)***	24 (33.8%) vs. 132 (19.9%)**
Cardiovascular disease	Yes	23 (12.6%) vs. 3 (0.5%)***	5 (7.0%) vs. 21 (3.2%)
**Feature of Deliveries**			
Gestational age (weeks)		33.2 (30.4–36.4) vs. 36.9 (34.3–38.6)***	33.2 ± 3.2 vs. 35.4 ± 4.3***
Delivery mode	vaginal delivery	2 (1.1%) vs. 57 (10.3%)***	0 (0%) vs. 59 (8.9%)***
	forceps delivery	1 (0.5%) vs. 2 (0.4%)	0 (0%) vs. 3 (0.5%)
	cesarean section	146 (80.2%) vs. 454 (82.4%)	65 (91.5%) vs. 535 (80.8%)
	(2nd-trimester) labor induction	20 (11.0%) vs. 30 (5.4%)	0 (0%) vs. 50 (7.6%)
	stillbirth delivery	13 (7.1%) vs. 8 (1.5%)	6 (8.5%) vs. 15 (2.3%)
**Feature of neonates**			
Neonatal death or stillbirth	Yes	33 (18.1%) vs. 38 (6.9%)***	6 (8.5%) vs. 65 (9.8%)
Admitted to NICU	Yes	101 (55.5%) vs. 201 (36.5%)***	52 (73.2%) vs. 250 (37.8%)***
Low birth weight	Yes	75 (41.2%) vs. 178 (32.3%)*	34 (47.9%) vs. 219 (33.1%)*
Birth weight of neonates (g)		1894.9 ± 975.8 vs. 2520.1 ± 993.7***	1810.5 ± 743.6 vs. 2431.8 ± 1039.1***
Apgar score (1 min)		8 (3–10) vs. 9 (9–10)***	8 (3–10) vs. 9 (8–10)**
Apgar score (5 min)		10 (7–10) vs. 10 (10–10)***	10 (8–10) vs. 10 (10–10)**
**Physical examination**			
Weight (kg)		77.6 ± 12.3 vs. 82. 9 ± 14.0***	76.9 ± 10.1 vs. 82.0 ± 13.9***
BMI		28.8 ± 4.0 vs. 30.7 ± 4.5***	28.6 ± 3.4 vs. 30.4 ± 4.5***
Systolic pressure (mmHg)		154.8 ± 28.4 vs. 148.4 ± 21.1**	152.3 ± 23.7 vs. 149.8 ± 23.4
Diastolic pressure (mmHg)		100.3 ± 20.8 vs. 95.7 ± 15.0**	99.6 ± 18.3 vs. 96.6 ± 16.5
**Laboratory examination**			
Leukocyte (× 10(9)/L)		11.56 ± 7.18 vs. 9.61 ± 3.17***	12.74 ± 10.40 vs. 9.80 ± 3.35*
Neutrophil (× 10(9)/L)		27.93 (7.35–75.78) vs. 12.76 (6.14–70.99)*	62.60 (9.13–78.40) vs. 14.51 (6.24–71.47)**
Platelet (× 10(9)/L)		149.7 ± 69.3 vs. 194.4 ± 64.7***	162.2 ± 58.9 vs. 184.7 ± 69.3**
APTT (s)		31.70 ± 9.16 vs. 29.89 ± 5.00*	32.19 ± 13.38 vs. 30.12 ± 5.03
Fbg (g/L)		4.00 ± 1.14 vs. 4.36 ± 1.60**	3.84 ± 1.20 vs. 4.30 ± 1.54*
ALT (U/L)		22.0 (16.6–35.3) vs. 17.0 (12.0–24.0)***	21.0 (15.0–28.0) vs. 18.0 (13.0–26.0)*
AST (U/L)		21.9 (14.0–34.5) vs. 17.0 (12.0–24.0)***	19.0 (11.0–28.0) vs. 18.0 (12.0–25.5)
Total protein (g/L)		53.7 ± 7.3 vs. 55.8 ± 7.0**	52.9 ± 7.3 vs. 55.6 ± 7.2**
Albumin (g/L)		28.9 ± 4.4 vs. 30.4 ± 4.7***	28.2 ± 4.2 vs. 30.2 ± 4.8**
Urea (mmol/L)		5.81 ± 2.60 vs. 4.43 ± 2.46***	5.83 ± 2.30 vs. 4.68 ± 2.59***
Creatinine (μmol/L)		71.0 ± 27.1 vs. 56.9 ± 14.8***	67.6 ± 20.5 vs. 59.9 ± 19.7**
Creatinine clearance rate		141.8 ± 53.8 vs. 176.5 ± 59.4***	141.3 ± 44.3 vs. 169.3 ± 61.8***
Uric acid (μmol/L)		418.0 ± 104.5 vs. 374.6 ± 99.9***	404.7 ± 86.1 vs. 383. 7 ± 105.1
Fasting blood-glucose (mmol/L)		4.76 ± 1.26 vs. 4.58 ± 1.16	4.36 ± 0.90 vs. 4.66 ± 1.22*
Serum sodium (mmol/L)		134.8 ± 14.4 vs. 137.2 ± 2.5*	134.2 ± 16.2 vs. 136.9 ± 5.9
Serum calcium (mmol/L)		1.97 ± 0.19 vs. 2.06 ± 0.17***	1.93 ± 0.24 vs. 2.04 ± 0.18***
Serum phosphorus (mmol/L)		1.37 ± 0.25 vs. 1.30 ± 0.24**	1.37 ± 0.19 vs. 1.32 ± 0.25
Urine pH		6.09 ± 0.64 vs. 6.26 ± 0.68**	6.23 ± 0.73 vs. 6.23 ± 0.67
Urine protein	negative	5 (2.7%) vs. 106 (19.2%)***	2 (2.8%) vs. 110 (16.6%)***
	(±)	10 (5.5%) vs. 54 (9.8%)	3 (4.2%) vs. 60 (9.1%)
	(+)	18 (9.9%) vs. 106 (19.2%)	6 (8.5%) vs. 120 (18.1%)
	(++)	53 (29.1%) vs. 124 (22.5%)	20 (28.2%) vs. 158 (23.9%)
	(+++)	72 (39.6%) vs. 120 (21.8%)	34 (47.9%) vs. 158 (23.9%)
	(++++)	24 (13.2%) vs. 41 (7.4%)	6 (8.5%) vs. 56 (8.5%)
Urine glucose	negative	161 (88.5%) vs. 519 (94.2%)*	64 (90.1%) vs. 618 (93.4%)
	(±)	16 (8.8%) vs. 14 (2.5%)	7 (9.9%) vs. 21 (3.2%)
	(+)	2 (1.1%) vs. 10 (1.8%)	0 (0%) vs. 12 (1.8%)
	(++)	3 (1.6%) vs. 5 (0.9%)	0 (0%) vs. 8 (1.2%)
	(+++)	0 (0%) vs. 2 (0.4%)	0 (0%) vs. 2 (0.3%)
	(++++)	0 (0%) vs. 1 (0.2%)	0 (0%) vs. 1 (0.2%)
Urine ketone	negative	171 (94.0%) vs. 490 (88.9%)*	69 (97.2%) vs. 593 (89.6%)*
	(±)	4 (2.2%) vs. 20 (3.6%)	0 (0%) vs. 24 (3.6%)
	(+)	2 (1.1%) vs. 6 (1.1%)	1 (1.4%) vs. 6 (0.9%)
	(++)	3 (1.6%) vs. 20 (3.6%)	1 (1.4%) vs. 22 (3.3%)
	(+++)	2 (1.1%) vs. 9 (1.6%)	0 (0%) vs. 11 (1.7%)
	(++++)	0 (0%) vs. 6 (1.1%)	0 (0%) vs. 6 (1.0%)
Urinary casts		1.7 (0.8–4.4) vs. 1.3 (0.1–3.7)**	2.0 (0.7–5.0) vs. 1.3 (0.1–3.8)
24-h urinary protein (mg)		3,29.2 (1,809.0–9380.0) vs. 1,860.0 (366.0–5,716.3)***	6,330.0 (2,206.9–10,370.0) vs. 2,061.5 (495.5–6,120.0)***
Cholesterol (mmol/L)		7.08 ± 2.13 vs. 6.70 ± 2.07*	7.24 ± 2.36 vs. 6.70 ± 2.03

* *P* < 0.05,

** *P* < 0.01,

*** *P* < 0.001.

**Figure 3:**
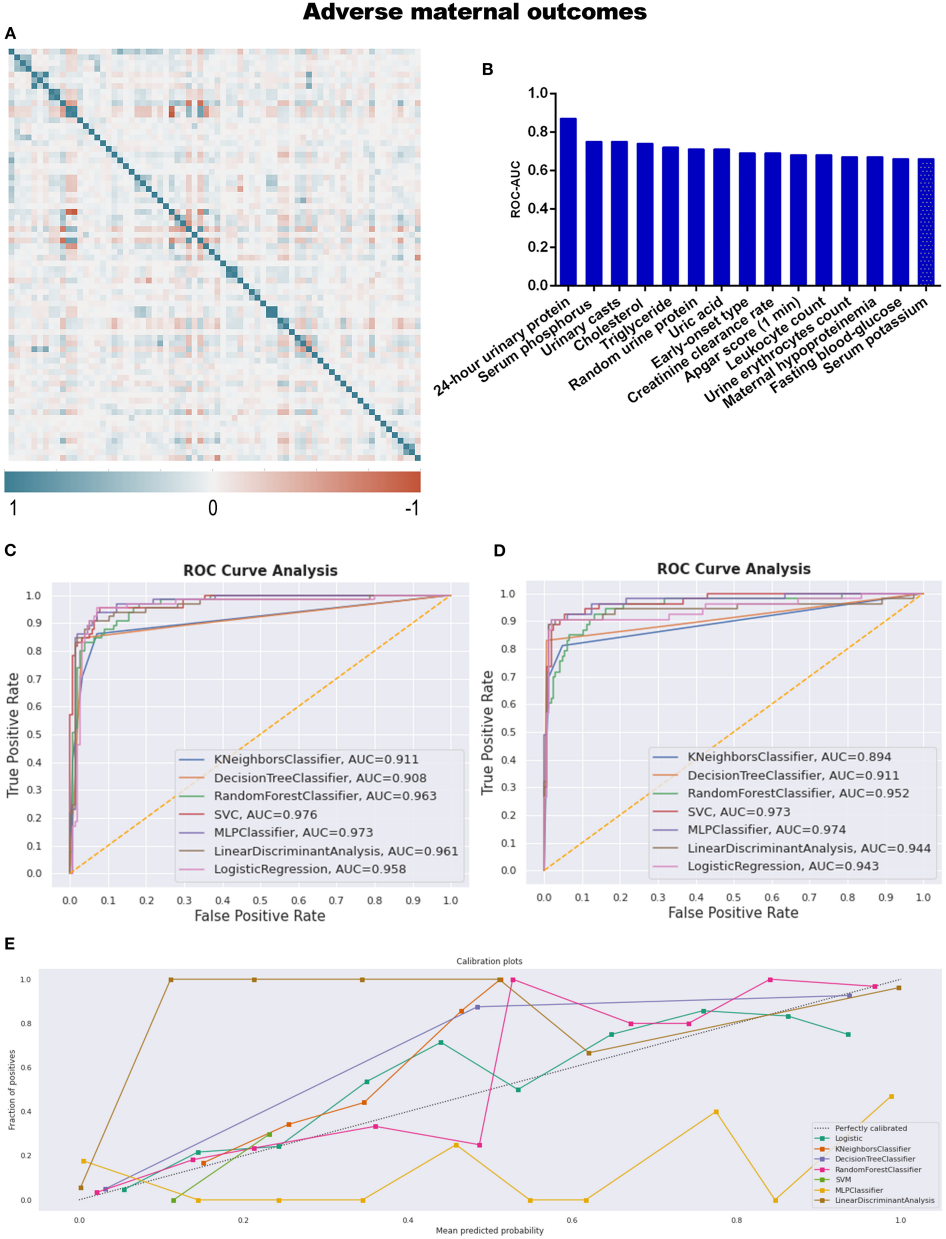
**(A)** The generated heat map according to correlation analysis to screen independent variables for adverse events; **(B)** feature selection of Top 15 adverse outcomes predictive variables according to the value of AUC calculated by DT algorithm; **(C)** the ROC-AUC values of machine learning and logistic regression predictive models developed from the imputed dataset; **(D)** the AUC values of models developed from the dataset without imputation; **(E)** the calibration curve generated from the models developed from imputed dataset.

The final target of this study is to compare the predictive efficiency of both logistic regression models and machine learning models in the field of pregnancy outcomes involving preeclampsia. Before developing the models, correlation analysis and feature selection of variables were performed. In Figure 3A, the generated heat map indicates the correlation relationship between variables. The sequence of variables along both the X and Y axes is identical to the sequence of variables in Supplementary Table 1. After removing dependent variables, independent variables were applied to DT models, and the AUC values of ROC were calculated and listed in Supplementary Table 3. The top 15 variables with higher AUC values are listed in Table 2 and Figure 3B.

**Table 2:** Feature selection of TOP 15 adverse outcomes predictive variables according to the value of AUC calculated by DT algorithm.

**Ranking**	**Maternal adverse outcome**		**Placenta abruption**		**Neonatal adverse outcomes**		**Low birth weight**	
	**Predictive variables**	**AUC**	**Predictive variables**	**AUC**	**Predictive variables**	**AUC**	**Predictive variables**	**AUC**
1	24-h urinary Protein	0.87	24-h urinary protein	0.86	Birth weight of neonates	0.90	24-h urinary protein	0.83
2	Serum phosphorus	0.75	Serum phosphorus	0.80	Triglyceride	0.86	Admitted to NICU	0.77
3	Urinary casts	0.75	Urinary casts	0.78	Urinary casts	0.83	Urine erythrocytes count	0.75
4	Cholesterol	0.74	Early-onset type	0.78	Early-onset type	0.83	Creatinine clearance rate	0.75
5	Triglyceride	0.72	Urine leukocytes count	0.77	24-h urinary Protein	0.82	Hemoglobin	0.74
6	Random urine protein	0.71	Hemoglobin	0.75	Cholesterol	0.82	Gestational age	0.73
7	Uric acid	0.71	Creatinine clearance rate	0.75	Urine erythrocytes count	0.78	Triglyceride	0.73
8	Early-onset type	0.69	Urine erythrocytes count	0.73	Neutrophil	0.77	Fbg	0.71
9	Creatinine clearance rate	0.69	Serum calcium	0.73	Creatinine clearance rate	0.76	Urinary casts	0.70
10	Apgar score (1 min)	0.68	Admitted to NICU	0.72	Albumin	0.73	Cholesterol	0.69
11	Leukocyte	0.68	Neutrophil	0.71	Apgar score (1 min)	0.72	Urine leukocytes count	0.69
12	Urine erythrocytes count	0.67	Random urine protein	0.69	APTT	0.72	APTT	0.68
13	Maternal hypoproteinemia	0.67	Triglyceride	0.69	Amniotic fluid index	0.71	Neutrophil	0.68
14	Fasting blood glucose	0.66	Cholesterol	0.69	Random urine protein	0.70	Early-onset type	0.68
15	Serum potassium	0.66	Fasting blood glucose	0.68	Serum phosphorus	0.70	ALT	0.67

After correlation analysis and feature selection, four variables (triglyceride, AUC = 0.72; urine erythrocytes count, AUC = 0.67; fasting blood-glucose, AUC = 0.66; and serum potassium, AUC = 0.66) demonstrated influential contribution on adverse maternal outcomes which declared no statistical significance between groups (Table 1; Figure 3B).

Different predictive models were developed from both imputed training datasets and training datasets without imputation. The AUC values were calculated to compare the discrimination efficiency when models were applied to testing datasets. The best-performing model referring to imputed datasets was SVM, with an AUC of 0.976; the sensitivity, specificity, positive predictive value (PPV), and negative predictive value (NPV) of SVM were 92.3, 92.3, 83.3, and 96.6%, respectively. The confusing matrices of different predictive models are listed in Supplementary Table 4. However, MLP ranked second (AUC = 0.973), as seen in Table 3 and Figure 3C. As to the models developed from datasets without imputations, the results were similar to those above, with MLP ranked first (AUC = 0.974) and SVM ranked second (AUC = 0.973; Figure 3D).

**Table 3:** The AUC, sensitivity, specificity, PPV, and NPV of different predictive models for adverse maternal outcomes.

	**With imputation**					**Without imputation**				
**Adverse maternal outcomes**	**AUC**	**SEN**	**SPE**	**PPV**	**NPV**	**AUC**	**SEN**	**SPE**	**PPV**	**NPV**
K-Nearest neighbor	0.911	0.708	0.968	0.902	0.888	0.894	0.698	0.988	0.949	0.912
Decision tree classifier	0.908	0.846	0.968	0.917	0.938	0.911	0.830	0.994	0.978	0.949
Random forest classifier	0.963	0.138	0.994	0.900	0.733	0.952	0.189	1.000	1.000	0.795
Support vector machine	**0.976**	0.923	0.923	0.833	0.966	0.973	0.849	0.988	0.957	0.954
Multi-layer perceptron	0.973	0.923	0.923	0.833	0.966	**0.974**	0.849	0.988	0.957	0.954
Linear discriminant analysis	0.961	0.831	0.987	0.964	0.933	0.944	0.830	0.988	0.957	0.948
Logistic regression	0.958	0.831	0.987	0.964	0.933	0.943	0.830	0.988	0.957	0.948
**Placental abruption**										
K-nearest neighbor	0.730	0.222	0.941	0.250	0.931	0.660	0.158	0.975	0.375	0.925
Decision tree classifier	0.594	0.222	0.906	0.174	0.929	0.555	0.105	0.896	0.087	0.914
Random forest classifier	0.795	0.000	1.000	0.000	0.918	**0.789**	0.000	1.000	0.000	0.914
Support vector machine	0.765	0.056	1.000	1.000	0.922	0.621	0.000	1.000	0.000	0.914
Multi-layer perceptron	**0.815**	0.056	1.000	1.000	0.922	0.772	0.050	1.000	1.000	0.914
Linear discriminant analysis	0.752	0.167	0.970	0.333	0.929	0.785	0.000	0.990	0.000	0.913
Logistic regression	0.764	0.167	0.970	0.333	0.929	0.779	0.000	0.990	0.000	0.913

AUC, area under the receiver operating characteristic curve; SEN, sensitivity; SPE, specificity; PPV, positive predictive value; NPV, negative predictive value.

“With imputation” indicates the predictive models developed from the imputed training dataset, while “Without imputation” indicates the models developed from the training dataset without imputation. The AUC values shown in bold indicate the best performed machine learning algorithm.

Not only did we prioritize discrimination skills, but we were also concerned with accurate probability judgment. According to the calibration curve in Figure 3E and Supplementary Figure 2A, whether the dataset was imputed or not, logistic regression was the recommended model for better probability estimation, as well as RF classifier.

**Figure 4:**
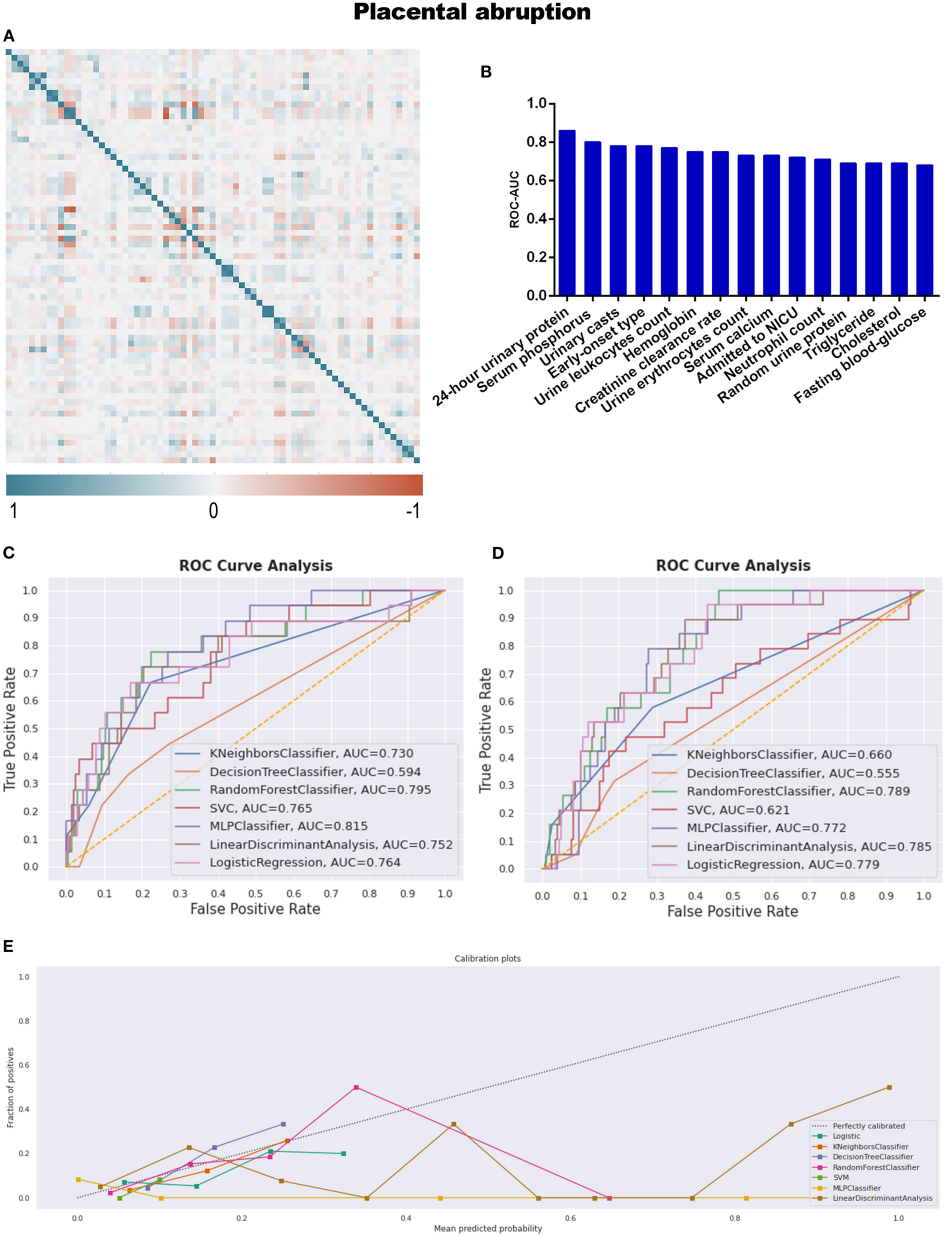
**(A)** The generated heat map according to correlation analysis to screen independent variables for adverse events; **(B)** feature selection of Top 15 adverse outcomes predictive variables according to the value of AUC calculated by DT algorithm; **(C)** the ROC-AUC values of machine learning and logistic regression predictive models developed from the imputed dataset; **(D)** the AUC values of models developed from the dataset without imputation; **(E)** the calibration curve generated from the models developed from imputed dataset. Referring to the adverse outcome of placental abruption.

Placental abruption, which is a more specific adverse maternal outcome indicating a severe emergency, was applied as a criterion for further grouping to explore more information. As described in Table 1 and Supplementary Table 5, twenty seven variables demonstrated statistical differences between PA and CON-PA, while another seven variables (serum phosphorus, urinary casts, urine leukocytes count, hemoglobin, urine erythrocytes count, triglyceride, and cholesterol) were selected as more predictive contributors depending on the feature selection process (Table 2; Figure 4B).

All predictive models for PA present lower levels of AUC values, ranging from 0.555 to 0.815, and MLP performed best under the imputed situation. The sensitivity, specificity, PPV, and NPV were 5.6, 100.0, 100.0, and 92.2%, respectively, in Table 3 and Figure 4C, while RF developed from the dataset without imputation demonstrated the best discriminative ability (Figure 4D). However, no model developed from this dataset with a skew distribution achieved satisfying calibration ability (Figure 4E; Supplementary Figure 2B).

### Adverse neonatal outcomes

**Figure 5:**
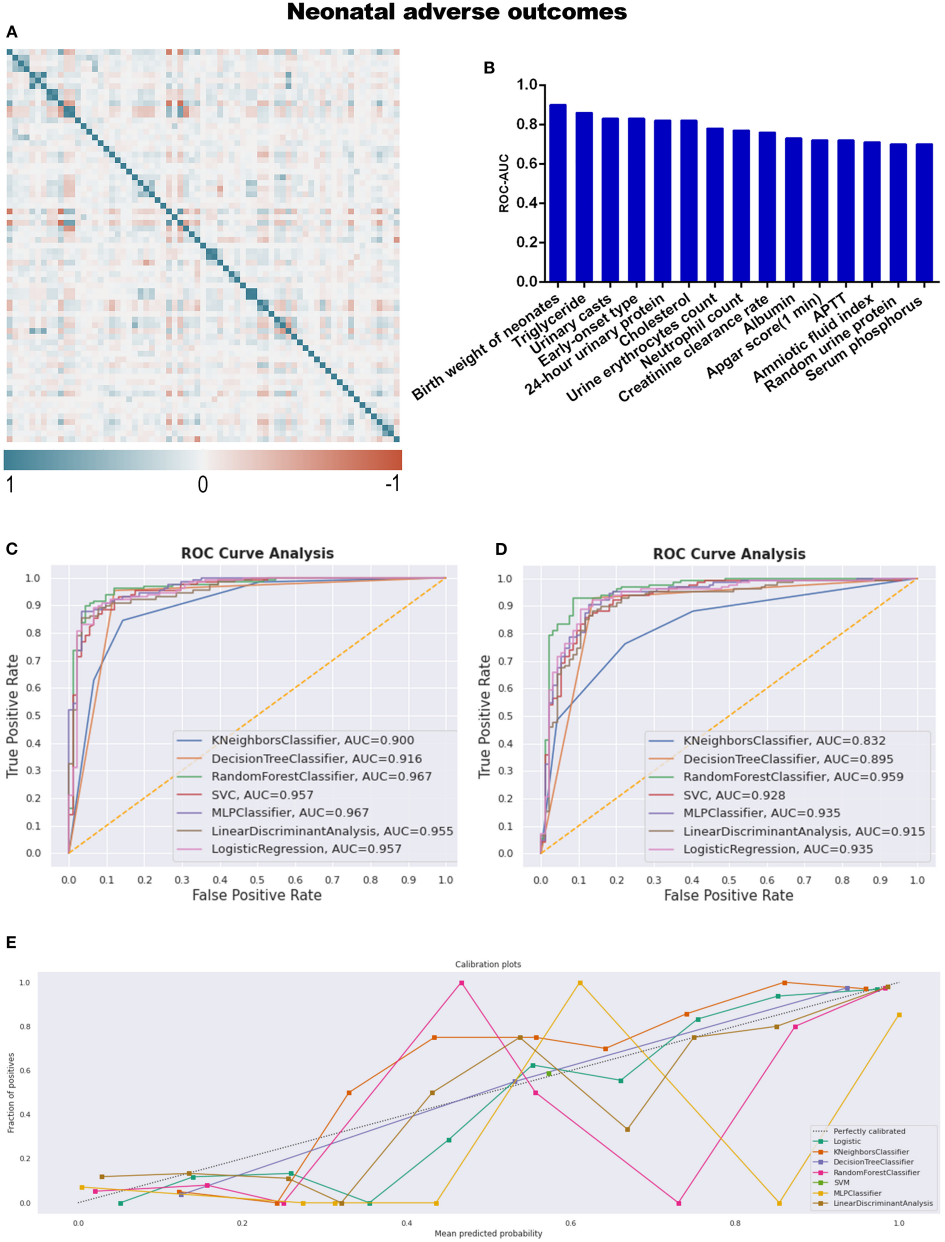
**(A)** The generated heat map according to correlation analysis to screen independent variables for adverse events; **(B)** feature selection of Top 15 adverse outcomes predictive variables according to the value of AUC calculated by DT algorithm; **(C)** the ROC-AUC values of machine learning and logistic regression predictive models developed from the imputed dataset; **(D)** the AUC values of models developed from the dataset without imputation; **(E)** the calibration curve generated from the models developed from imputed dataset. Referring to an adverse neonatal outcome.

Referring to adverse neonatal outcomes, we observed that an amniotic fluid index is a characteristic variable involving adverse outcomes compared to adverse maternal outcomes (Table 4; Supplementary Table 6). Under the same workflow mentioned above, triglyceride and APTT are the missing informative items neglected by statistical analysis while responding during preprocessing steps before model construction, as seen in Table 2 and Figure 5B.

**Table 4:** The AUC, sensitivity, specificity, PPV, and NPV of different predictive models for adverse neonatal outcomes.

	**With imputation**					**Without imputation**				
**Adverse neonatal outcomes**	**AUC**	**SEN**	**SPE**	**PPV**	**NPV**	**AUC**	**SEN**	**SPE**	**PPV**	**NPV**
K-nearest neighbor	0.900	0.845	0.857	0.893	0.796	0.832	0.762	0.777	0.821	0.709
Decision tree classifier	0.916	0.953	0.879	0.918	0.930	0.895	0.929	0.862	0.900	0.900
Random forest classifier	**0.967**	0.938	0.879	0.917	0.909	**0.959**	0.929	0.883	0.914	0.902
Support Vector Machine	0.957	0.922	0.868	0.908	0.888	0.928	0.952	0.851	0.896	0.930
Multi-Layer Perceptron	**0.967**	0.922	0.868	0.908	0.888	0.935	0.952	0.851	0.896	0.930
Linear Discriminant Analysis	0.955	0.907	0.879	0.914	0.870	0.915	0.873	0.862	0.894	0.835
Logistic regression	0.957	0.907	0.879	0.914	0.870	0.935	0.873	0.862	0.894	0.835
**Low birth weight**										
K-Nearest Neighbor	0.793	0.590	0.852	0.687	0.791	0.782	0.628	0.831	0.671	0.803
Decision tree classifier	0.862	0.705	0.901	0.797	0.848	**0.904**	0.808	0.915	0.840	0.897
Random forest classifier	0.893	0.423	0.937	0.786	0.747	0.866	0.551	0.873	0.705	0.780
Support Vector Machine	0.919	0.718	0.908	0.812	0.854	0.879	0.705	0.789	0.647	0.830
Multi-Layer Perceptron	0.914	0.718	0.908	0.812	0.854	0.869	0.705	0.789	0.647	0.830
Linear Discriminant Analysis	0.918	0.782	0.873	0.772	0.879	0.861	0.667	0.838	0.693	0.821
Logistic regression	**0.925**	0.782	0.873	0.772	0.879	0.857	0.667	0.838	0.693	0.821

The AUC values shown in bold indicate the best performed machine learning algorithm.

RF (sensitivity: 93.8%, specificity: 87.9%, PPV: 91.7%, NPV: 90.9%), and MLP were the top two discriminative models when the dataset was imputed, with AUC values of 0.967 (Table 4; Figure 5C); meanwhile, RF was also the best model for discrimination as to the dataset without imputation (Figure 5D). The qualitative results obtained from calibration curves revealed that the DT demonstrated optimal calibration, whether imputation happened or not, which can be seen in Figure 5E and Supplementary Figure 2C.

**Figure 6:**
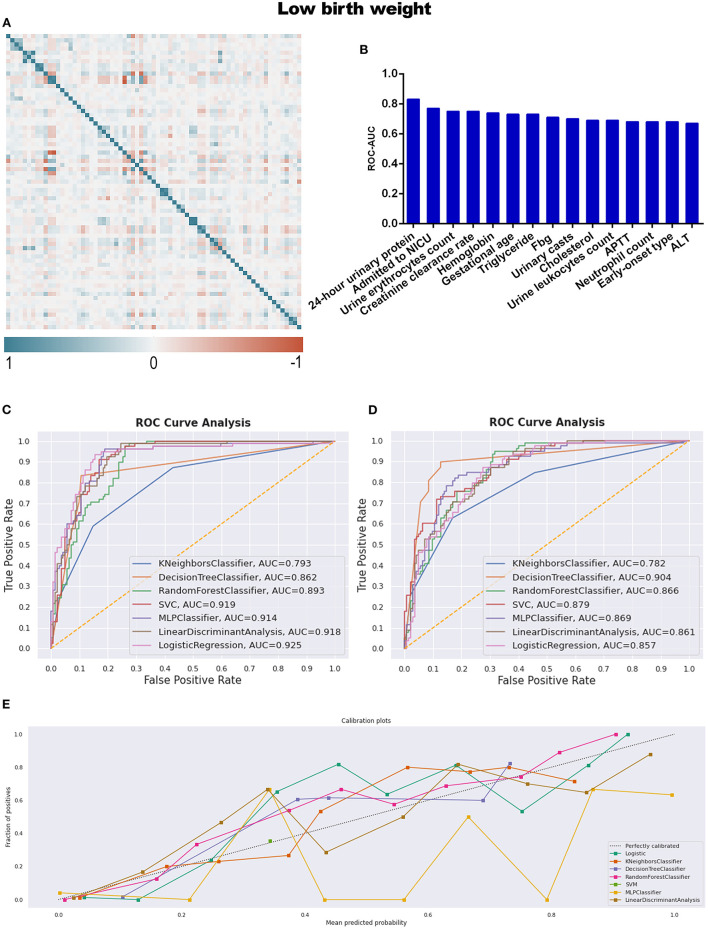
**(A)** The generated heat map according to correlation analysis to screen independent variables for adverse events; **(B)** feature selection of Top 15 adverse outcomes predictive variables according to the value of AUC calculated by DT algorithm; **(C)** the ROC-AUC values of machine learning and logistic regression predictive models developed from the imputed dataset; **(D)** the AUC values of models developed from the dataset without imputation; **(E)** the calibration curve generated from the models developed from imputed dataset. Referring to the adverse outcome of low birth weight.

With respect to instances of low birth weight neonates, LBW and CON-LBW were grouped to explore more information under our current data processing procedures, similarly to the grouping idea of placental abruption. The interesting male advantage, that male neonates have lower rates of low birth weight, was established when gender differences were analyzed with statistical significance between the two groups, as listed in Table 5 and Supplementary Table 7. APTT is the only variable excluded by the statistical method and included in the TOP 15 contributing factors screened by feature selection (Table 2; Figure 6B).

**Table 5:** Variables with statistical significance between adverse neonatal outcomes group and control group.

**Variables**		**Adverse neonatal outcomes**	**Low birth weight**
		**ANO vs. CON-ANO**	**LBW vs. CON-LBW**
**Demography**			
Gravidity		2 (1–3) vs. 2 (1–2)**	2 (1–3) vs. 2 (1–3)
Parity		0 (0–1) vs. 0 (0–1)**	0 (0–1) vs. 0 (0–1)
**Complications**			
Thyroid disease	Yes	48 (11.3%) vs. 15 (4.8%)**	33 (13.0%) vs. 30 (6.3%)**
Twin pregnancy	Yes	18 (4.3%) vs. 11 (3.5%)	2 (0.8%) vs. 27 (5.6%)**
Early-onset type	Yes	268 (63.4%) vs. 8 (2.6%)***	–
Maternal hypoproteinemia	Yes	127 (30.0%) vs. 29 (9.4%)***	79 (31.2%) vs. 77 (16.0%)***
Thrombocytopenia	Yes	53 (12.5%) vs. 7 (2.3%)***	27 (10.7%) vs. 32 (6.7%)
Impaired liver function	Yes	34 (8.0%) vs. 4 (1.3%)***	16 (6.3%) vs. 21 (4.4%)
Cardiovascular disease	Yes	20 (4.7%) vs. 6 (1.9%)*	11 (4.3%) vs. 14 (2.9%)
Renal insufficiency	Yes	24 (5.7%) vs. 1 (0.3%)***	16 (6.3%) vs. 25 (5.2%)
Placental abruption	Yes	61 (14.4%) vs. 10 (3.2%)***	34 (13.4%) vs. 37 (7.7%)*
HELLP syndrome	Yes	36 (8.5%) vs. 5 (1.6%)***	9 (3.6%) vs. 17 (3.5%)
**Feature of deliveries**			
Gestational age (weeks)		32.9 ± 4.0 vs. 38.3 ± 1.9***	34.0 (31.4–36.1) vs. 37.3 (34.9–39.0)***
Delivery mode	vaginal delivery	14 (3.3%) vs. 45 (14.5%)***	7 (2.8%) vs. 52 (10.8%)***
	forceps delivery	0 (0%) vs. 3 (1.0%)	0 (0%) vs. 3 (0.6%)
	cesarean section	338 (79.9%) vs. 262 (84.5%)	222 (87.7%) vs. 378 (78.8%)
	(2nd-trimester) labor induction	50 (11.8%) vs. 0 (0%)	20 (7.9%) vs. 30 (6.3%)
	stillbirth delivery	21 (5.0%) vs. 0 (0%)	4 (1.6%) vs. 17 (3.5%)
**Feature of neonates**			
Gender of neonates	Male	188 (44.4%) vs. 152 (49.0%)	102 (40.3%) vs. 238 (49.6%)*
Admitted to NICU	Yes	–	179 (70.8%) vs. 123 (25.6%)***
Birth weight of neonates (g)		1,736.3 ± 777.2 vs. 3,247.9 ± 627.4***	1,547.8 ± 501.1 vs. 2814.9 ± 974.6***
Apgar score (1 min)		8 (5–10) vs. 10 (9–10)***	9 (7–10) vs. 9 (9–10)***
Apgar score (5 min)		10 (8–10) vs. 10 (10–10)***	10 (9–10) vs. 10 (10–10)**
**Physical examination**			
Weight (kg)		79.6 ± 12.2 vs. 83.8 ± 15.6***	78.8 ± 11.5 vs. 82.9 ± 14.8***
BMI		29.5 ± 3.9 vs. 31.1 ± 5.1***	29.1 ± 3.6 vs. 30.7 ± 4.8***
Systolic pressure (mmHg)		152.8 ± 25.0 vs. 146.3 ± 20.4***	152.6 ± 23.5 vs. 148.7 ± 23.2*
Diastolic pressure (mmHg)		98.4 ± 18.3 vs. 94.8 ± 14.0**	98.2 ± 17.3 vs. 96.2 ± 16.4
**Laboratory examination**			
Leukocyte (× 10(9)/L)		10.58 ± 3.76 vs. 9.48 ± 5.36**	9.70 (7.96–11.53) vs. 9.24 (7.48–11.26)
Neutrophil (× 10(9)/L)		63.00 (8.44–74.93) vs. 7.18 (5.52–51.60)***	59.80(7.96–74.67) vs. 9.28 (5.94–69.74)***
Hemoglobin (g/L)		122.5 ± 22.4 vs. 118.3 ± 15.1**	125.4 ± 20.8 vs. 118.4 ± 18.6***
Hematokrit (%)		36.9 ± 6.9 vs. 36.0 ± 4.8*	37.6 ± 6.8 vs. 35.9 ± 5.5***
Platelet (× 10(9)/L)		172.2 ± 75.0 vs. 197.9 ± 55.3***	176.7 ± 78.0 vs. 186.6 ± 62.9
PT (s)		10.66 ± 1.45 vs. 11.83 ± 7.92**	10.55 ± 1.56 vs. 11.47 ± 6.43*
Fbg (g/L)		4.11 ± 1.42 vs. 4.55 ± 1.59***	4.05 ± 1.52 vs. 4.42 ± 1.48**
ALT (U/L)		21.0 (16.0–32.0) vs. 14.0 (10.0–20.0)***	22.0 (16.5–33.0) vs. 17.0 (11.0–23.0)***
AST (U/L)		18.0 (12.0–30.0) vs. 17.0 (12.0–22.1)*	19.0 (13.0–30.0) vs. 17.0 (12.0–24.0)**
Total protein (g/L)		53.13 ± 7.29 vs. 58.15 ± 6.24***	53.27 ± 6.87 vs. 56.24 ± 7.28***
Albumin (g/L)		28.62 ± 4.57 vs. 31.90 ± 4.19***	28.79 ± 4.17 vs. 30.65 ± 4.84***
Globulin (g/L)		24.56 ± 5.16 vs. 27.81 ± 28.53*	24.43 ± 5.02 vs. 26.69 ± 23.19
Urea (mmol/L)		5.43 ± 3.02 vs. 3.93 ± 1.38***	5.47 ± 2.14 vs. 4.44 ± 2.71***
Creatinine (μmol/L)		64.90 ± 22.16 vs. 54.35 ± 12.29***	64.35 ± 19.99 vs. 58.41 ± 18.66***
Creatinine clearance rate		151.76 ± 54.15 vs. 185.90 ± 64.35***	150.87 ± 54.68 vs. 175.04 ± 63.49***
Uric Acid (μmol/L)		407.77 ± 106.88 vs. 349.90 ± 89.27***	419.24 ± 114.77 vs. 364.08 ± 91.59***
Serum sodium (mmol/L)		136.17 ± 9.67 vs. 137.29 ± 2.76*	136.17 ± 8.79 vs. 136.91 ± 6.85
Serum calcium (mmol/L)		2.01 ± 0.20 vs. 2.07 ± 0.15***	2.01 ± 0.19 vs. 2.05 ± 0.18**
Serum phosphorus (mmol/L)		1.36 ± 0.26 vs. 1.26 ± 0.20***	1.40 ± 0.27 vs. 1.28 ± 0.21***
Urine leukocytes count		22.24 (2.10–65.05) vs. 2.78 (1.00–15.00)***	21.68 (2.00–54.49) vs. 4.65 (1.00–31.14)***
Urine protein	negative	25 (5.9%) vs. 83 (26.8%)***	20 (7.9%) vs. 88 (18.3%)***
	(±)	20 (4.7%) vs. 43 (13.9%)	6 (2.4%) vs. 57 (11.9%)
	(+)	53 (12.5%) vs. 72 (23.2%)	33 (13.0%) vs. 92 (19.2%)
	(++)	125 (29.6%) vs. 57 (18.4%)	72 (28.5%) vs. 110 (22.9%)
	(+++)	152 (35.9%) vs. 42 (13.5%)	91 (36.0%) vs. 103 (21.5%)
	(++++)	48 (11.3%) vs. 13 (4.2%)	31 (12.3%) vs. 30 (6.3%)
Urine erythrocytes count		17.79 (6.67–38.92) vs. 1.98 (0.00–11.12)***	16.68 (4.78–32.25) vs. 6.67 (0.45–23.91)***
Urine ketone	negative	389 (92.0%) vs. 270 (87.1%)*	238 (94.1%) vs. 420 (87.5%)**
	(±)	13 (3.1%) vs. 12 (3.9%)	5 (2.0%) vs. 20 (4.2%)
	(+)	4 (0.9%) vs. 4 (1.3%)	3 (1.2%) vs. 5 (1.0%)
	(++)	14 (3.3%) vs. 12 (3.8%)	5 (2.0%) vs. 22 (4.6%)
	(+++)	1 (0.2%) vs. 9 (2.9%)	0 (0%) vs. 9 (1.9%)
	(++++)	2 (0.5) vs. 3 (0.9%)	2 (0.8%) vs. 4 (0.8%)
Urinary casts		1.97 (0.90–5.12) vs. 0.88 (0.00–2.36)***	2.15 (0.88–5.38) vs. 1.00 (0.00–2.77)***
24-h urinary protein (mg)		5,520.0 (2,063.8–9,820.0) vs. 675.6 (236.6–1984.5)***	5,064.7 (1,960.2–10,940.0) vs. 1,380.0 (350.0–4,820.4)***
Cholesterol (mmol/L)		7.12 ± 2.46 vs. 6.37 ± 1.28***	7.37 ± 2.78 vs. 6.47 ± 1.50***
Triglyceride (mmol/L)		4.57 ± 2.94 vs. 3.89 ± 1.32***	4.62 ± 3.00 vs. 4.08 ± 2.01*
Ultrasonic examination			
Amniotic fluid index (cm)		5.7 ± 3.1 vs. 8.3 ± 3.9***	5.7 ± 3.2 vs. 7.3 ± 3.8***

* *P* < 0.05,

** *P* < 0.01,

*** *P* < 0.001.

The identified best discriminative model for an imputed dataset is the Logistic Regression model, reporting an AUC of 0.925. The sensitivity, specificity, PPV, and NPV were 78.2, 87.3, 77.2, and 87.9%, respectively, in Table 4 and Figure 6C; referring to the dataset without imputation, DT was the best one, as shown in Figure 6D. RF is the best calibration model for an imputed dataset, while DT performed best relating to the dataset without imputation (Figure 6E; Supplementary Figure 2D).

## Discussion

Machine learning methods were applied in three procedures in our research: the imputation of missing values, the feature selection of variables, and the development of predictive models. Missing data is an inevitable and challenging issue in our retrospective study, which may lead to a biased conclusion if handled inappropriately. The K-nearest neighbors rule is an effective algorithm to impute missing data (34), although it should not be the fundamental solution. The reasons for missing data are probably because (1) the clinical significance of a series of laboratory indicators was not evidenced sufficiently as the biomarkers to predict adverse outcomes of preeclampsia. Therefore, the lack of standard clinical examination procedures limits clinicians from collecting data according to a unified scheme; (2) the emergencies of clinical practice prevent practitioners from collecting required indicators in time. For the above reasons, more research is still needed to investigate the relationship between reliable biomarkers and adverse outcomes persuasively based on big medical data. The development of efficient predictive models may promote the avoidance of most emergencies.

The feature selection process, which employs the DT machine learning model and is evaluated by the AUC of ROC, provides a novel screening strategy for influential candidate variables. Some screened variables may be neglected if statistical significance is the only selection criteria. However, there was a significantly lower level of AUC values referring to all the predictive models for placental abruption and unsatisfactory calibration ability. Indeed, placental abruption is not only challenging but also a daunting task for obstetricians. The variables we collected from a routine prenatal examination may not be sufficiently influential predictors for this emergency issue. Seeking for a definite indicator, as well as assessing the influence of individual variables on the prediction of this adverse outcome, which depends on the explainable feature selection techniques, for instance, SHAP (SHapley Additive exPlanations) (35), probably is a promising research tendency besides traditional statistics.

The idea of “statistical significance should retire” is not what we are appealing to. Instead, clinicians would better abandon the exaggerated criticism about statistics, be alert to the booming ML domain, realize the relevance between traditional biostatistics and machine learning algorithms, explore the characteristics of different strategies and facilitate the combined benefits for clinical practices. Overall, MLP, RF, and SVM demonstrated better discriminative ability when the models were developed from imputed datasets to predict adverse pregnancy outcomes. In addition, LR was the best discriminator for low birth weight; while RF, MLP, and DT discriminated against populations with unsatisfactory outcomes better by referring to the original datasets with missing values; moreover, DT, RF, and LR demonstrated more accurate probability estimation according to the calibration curves regardless of whether imputation was proposed or not. This research provided evidence of modeling preference when adverse pregnancy outcomes were predicted. However, the models are only preferred depending on the current datasets they were trained on. A larger number of medical records is still needed for further evaluation between the LR and ML models.

Although we still cannot reveal the definite reasons for all the results we achieved (36), it remains a promising direction for clinical prediction. Predictions with a long time span may have low accuracy, while the separated short-term predictions of different events will be combined as a customized predictive package for individual risk assessment. Limited work has been done on real-time automated predictive models that could be embedded in an electronic medical record system. Using alert thresholds, we observed that the onset of preeclampsia in a population with high risk in pre-pregnancy or early pregnancy and the susceptibility of involved women to adverse outcomes in late pregnancy could all be flagged timely. Beneficial interventions could be conducted for the well-being of patients (37). Taken together, this is what we are aiming for.

As mentioned above, validating the reasonable applied range of statistical and machine learning techniques seems to be a more appropriate way forward, not relying solely on statistical significance or overusing machine learning inappropriately. Medical statistics have come a long way, and combining statistics and machine learning is a long way to go with the interdisciplinary cooperative efforts.

## Conclusion

Statistical analysis and machine learning are two scientific domains sharing similar themes, while the modeling procedures for predictive models developed by the two domains still demonstrate various characteristics. The influential variables screened by preprocessing steps did not overlap with those determined by statistical differences. Moreover, MLP, RF, and SVM performed better discriminative power for prediction overall, while DT, RF, and LR yielded better calibration capability. Future work will focus on accumulating more evidence about applying different algorithmic predictive models to structural medical records. The long-term goal is to combine a series of real-time predictive models as chronological predictive packages embedded in electronic medical record systems to alarm the adverse situations automatically and effectively.

## Data availability statement

The raw data supporting the conclusions of this article will be made available by the authors, without undue reservation.

## Ethics statement

The Shengjing Hospital of China Medical University Ethics Review Board, reviewed and approved the study on March 4, 2013 (2013PS68K). The Second Affiliated Hospital of Dalian Medical University Ethics Review Board approved the study on May 9, 2022 (2022-033). Requirement for written informed consent was waived by the review board because of the retrospective, de-identified nature of the patient data. Written informed consent for participation was not required for this study in accordance with the national legislation and the institutional requirements.

## Author contributions

DZ, XH, TH, and FC devised the study plan. FL, NX, and FK helped with data acquisition. LW contributed to the interpretation of data. DZ and XH built the dataset, analyzed the data, and drafted the article. MK completed language editing and revised the draft. KS and CQ supervised the research. All authors read the draft manuscript and made significant intellectual contributions to the final version.

## Conflict of interest

The authors declare that the research was conducted in the absence of any commercial or financial relationships that could be construed as a potential conflict of interest.

## Publisher's note

All claims expressed in this article are solely those of the authors and do not necessarily represent those of their affiliated organizations, or those of the publisher, the editors and the reviewers. Any product that may be evaluated in this article, or claim that may be made by its manufacturer, is not guaranteed or endorsed by the publisher.

## Supplementary material

Supplementary Figure 1 The comparison of different imputation strategies.

Supplementary Figure 2 The calibration curves are generated from the models developed from the dataset without imputation.

Supplementary Table 1 Missing data rate.

Supplementary Table 2 Statistical description and test of variables between the adverse maternal outcomes group and the control group.

Supplementary Table 3 ROC-AUC of predictive variables calculated by DT classifier.

Supplementary Table 4 Confusing matrixes of predictive models.

Supplementary Table 5 Statistical description and test of variables between the placental abruption and control groups.

Supplementary Table 6 Statistical description and test of variables between the adverse neonatal outcomes group and the control group.

Supplementary Table 7 Statistical description and test of variables between the low birth weight group and the control group.
