# Supplementary figures and images for: Comparison of machine learning and logistic regression as predictive models for adverse maternal and neonatal outcomes of preeclampsia: A retrospective study
Source: Front Cardiovasc Med. 2022 Oct 12;9:959649. doi: 10.3389/fcvm.2022.959649 (PMC9596815; doi:10.3389/fcvm.2022.959649)

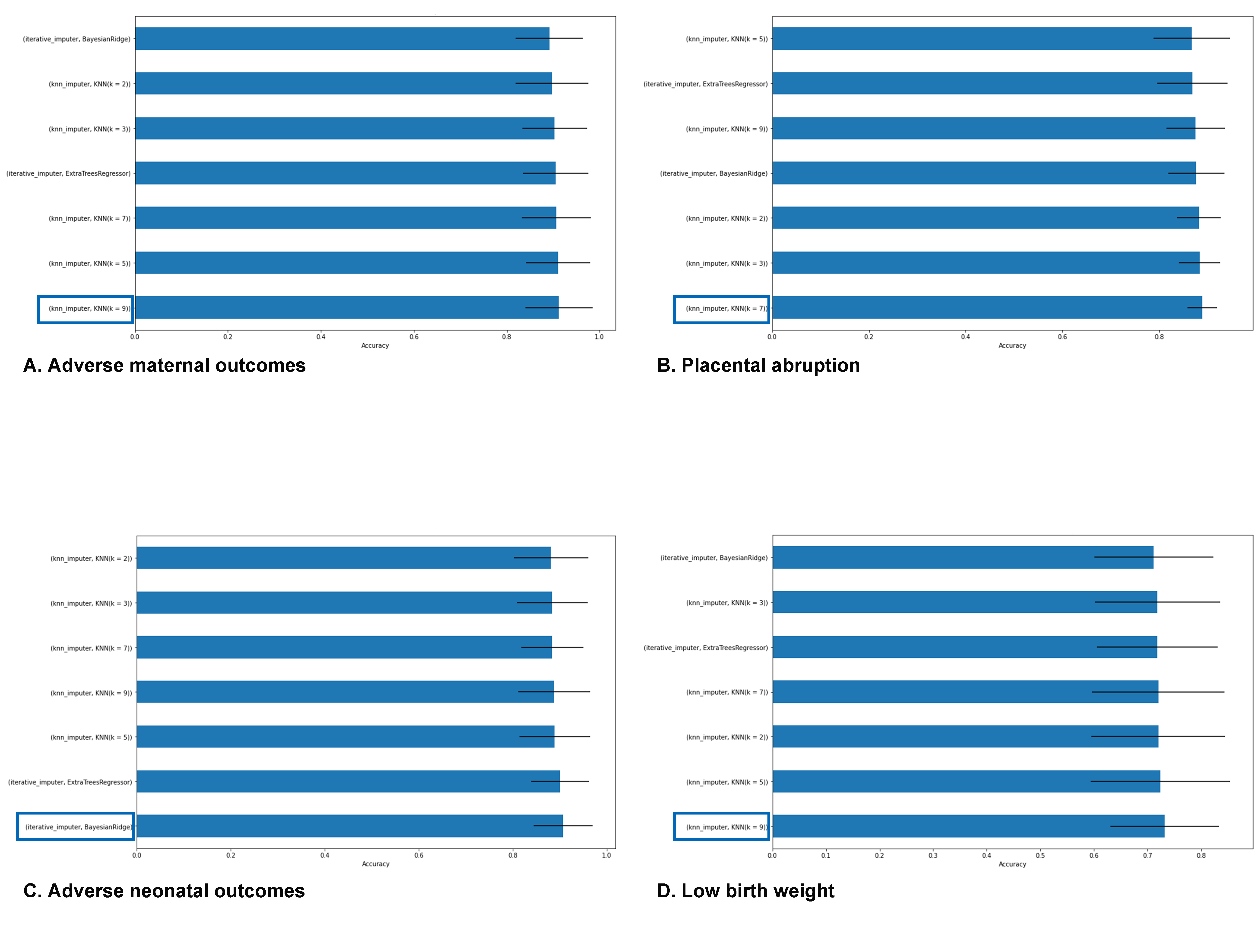

Supplement: Supplementary Figure 1 — The comparison of different imputation strategies. [file Image_1.TIF]

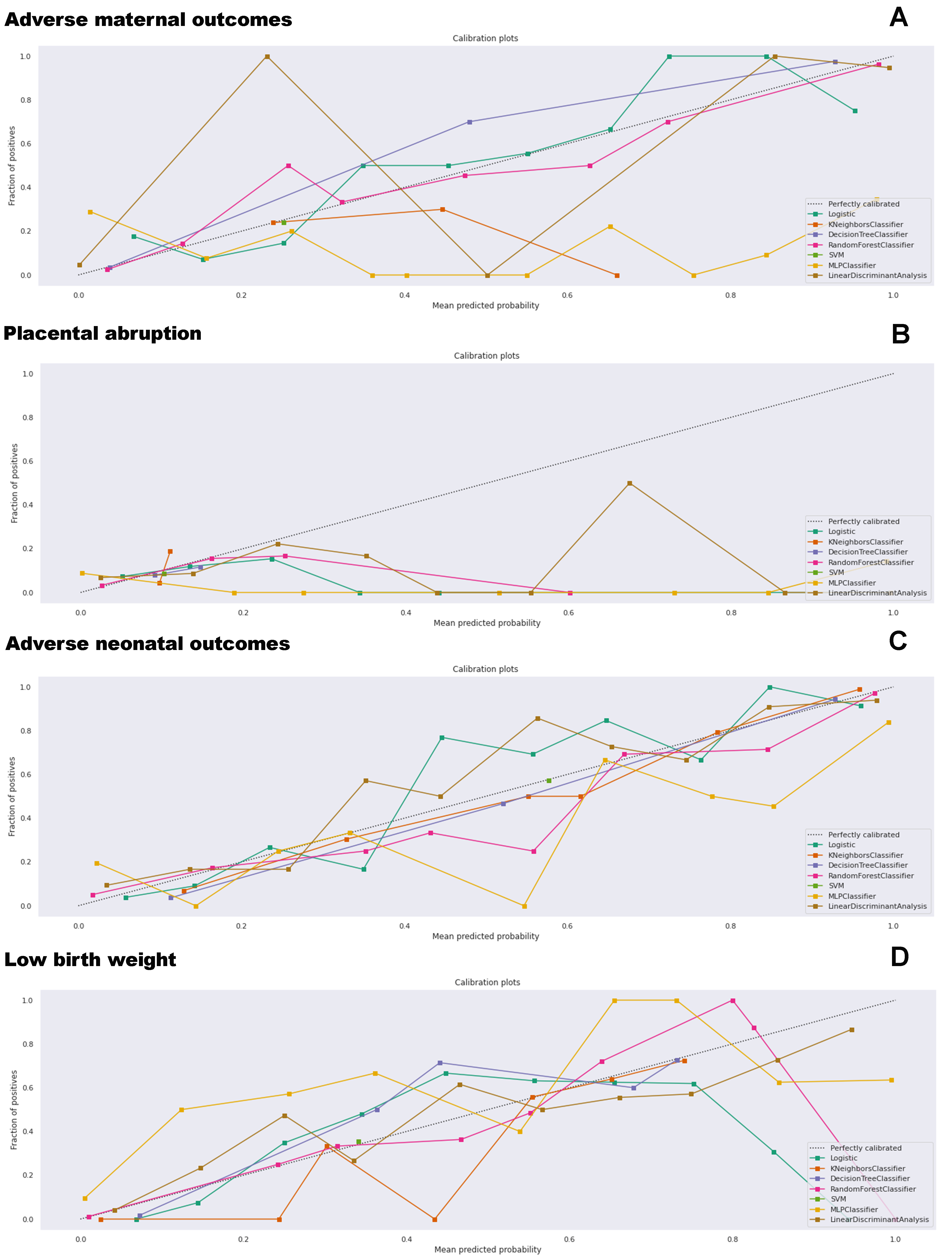

Supplement: Supplementary Figure 2 — The calibration curves are generated from the models developed from the dataset without imputation. [file Image_2.tif]
