# Supplementary material for: Comparison of machine learning and logistic regression as predictive models for adverse maternal and neonatal outcomes of preeclampsia: A retrospective study
Source: Front Cardiovasc Med. 2022 Oct 12;9:959649. doi: 10.3389/fcvm.2022.959649 (PMC9596815; doi:10.3389/fcvm.2022.959649)
Supplement: Supplementary Table 2 — Statistical description and test of variables between the adverse maternal outcomes group and the control group. [file Table_2.DOCX]

**Supplementary 2. Statistical description and test of variables between the adverse maternal outcomes group and the control group.**

| Variables |  | Study group | Control group | *P* value |
| --- | --- | --- | --- | --- |
| DEMOGRAPHY |  |  |  |  |
| Age (years) |  | 31.7±5.3 | 31.1±5.2 | 0.123 |
| Age over 35 | Yes | 13 (7.1%) | 36 (6.5%) | 0.775 |
| **Gravidity** |  | 2 (1-3) | 2 (1-3) | <0.05 |
| Parity |  | 0 (0-1) | 0 (0-1) | 0.158 |
| COMPLICATIONS |  |  |  |  |
| Chronic Hypertension | Yes | 26 (14.3%) | 71 (12.9%) | 0.629 |
| Diabetes (Pregestational or Gestational) | Yes | 30 (16.5%) | 122 (22.1%) | 0.103 |
| Thyroid Disease | Yes | 13 (7.1%) | 50 (9.1%) | 0.420 |
| IVF-ET | Yes | 4 (2.2%) | 16 (2.9%) | 0.612 |
| Scarred Uterus | Yes | 26 (14.3%) | 71 (12.9%) | 0.629 |
| Twin Pregnancy | Yes | 3 (1.6%) | 26 (4.7%) | 0.065 |
| **Early-onset type** | Yes | 117 (64.3%) | 159 (28.9%) | <0.001 |
| **Maternal Hypoproteinemia** | Yes | 70 (38.5%) | 86 (15.6%) | <0.001 |
| **Cardiovascular disease** | Yes | 23 (12.6%) | 3 (0.5%) | <0.001 |
| FEATURE OF DELIVERIES |  |  |  |  |
| **Gestational Age (weeks)** |  | 33.2 (30.4-36.4) | 36.9 (34.3-38.6) | <0.001 |
| **Delivery Mode** | vaginal delivery | 2 (1.1%) | 57 (10.3%) | <0.001 |
|  | forceps delivery | 1 (0.5%) | 2 (0.4%) |  |
|  | cesarean section | 146 (80.2%) | 454 (82.4%) |  |
|  | 2nd-trimester labor induction | 20 (11.0%) | 30 (5.4%) |  |
|  | stillbirth delivery | 13 (7.1%) | 8 (1.5%) |  |
| FEATURE OF NEONATES |  |  |  |  |
| Gender of Neonates | Male | 89 (48.9%) | 257 (46.6%) | 0.597 |
| **Neonatal Death or Stillbirth** | Yes | 33 (18.1%) | 38 (6.9%) | <0.001 |
| **Admitted to NICU** | Yes | 101 (55.5%) | 201 (36.5%) | <0.001 |
| **Low Birth Weight** | Yes | 75 (41.2%) | 178 (32.3%) | <0.05 |
| **Birth Weight of Neonates (g)** |  | 1894.9±975.8 | 2520.1±993.7 | <0.001 |
| **Apgar Score (1 min)** |  | 8 (3-10) | 9 (9-10) | <0.001 |
| **Apgar Score (5 min)** |  | 10 (7-10) | 10 (10-10) | <0.001 |
| PHYSICAL EXAMINATION |  |  |  |  |
| **Weight (kg)** |  | 77.6±12.3 | 82. 9±14.0 | <0.001 |
| Height (cm) |  | 163.9±4.1 | 164.1±4.4 | 0.587 |
| **BMI** |  | 28.8±4.0 | 30.7±4.5 | <0.001 |
| **Systolic Pressure (mmHg)** |  | 154.8±28.4 | 148.4±21.1 | <0.01 |
| **Diastolic Pressure (mmHg)** |  | 100.3±20.8 | 95.7±15.0 | <0.01 |
| LABORATORY EXAMINATION |  |  |  |  |
| **Leukocyte ( × 10(9)/L)** |  | 11.56±7.18 | 9.61±3.17 | <0.001 |
| **Neutrophil ( × 10(9)/L)** |  | 27.93 (7.35-75.78) | 12.76 (6.14-70.99) | <0.05 |
| Erythrocyte ( × 10(12)/L) |  | 3.88±0.65 | 4.24±5.18 | 0.354 |
| Hemoglobin (g/L) |  | 118.9±23.0 | 121.6±17.9 | 0.162 |
| Hematokrit (%) |  | 35.8±7.7 | 36.8±5.3 | 0.127 |
| **Platelet ( × 10(9)/L)** |  | 149.7±69.3 | 194.4±64.7 | <0.001 |
| PT (s) |  | 11.27±1.61 | 11.11±6.04 | 0.723 |
| **APTT (s)** |  | 31.70±9.16 | 29.89±5.00 | <0.05 |
| **Fbg (g/L)** |  | 4.00±1.14 | 4.36±1.60 | <0.01 |
| TT (s) |  | 16.53±1.79 | 16.07±5.89 | 0.305 |
| **ALT (U/L)** |  | 22 (16.6-35.3) | 17 (12-24) | <0.001 |
| **AST (U/L)** |  | 21.9 (14-34.5) | 17 (12-24) | <0.001 |
| **Total Protein (g/L)** |  | 53.7±7.3 | 55.8±7.0 | <0.01 |
| **Albumin (g/L)** |  | 28.9±4.4 | 30.4±4.7 | <0.001 |
| Globulin (g/L) |  | 25.0±4.4 | 26.2±21.8 | 0.452 |
| **Urea (mmol/L)** |  | 5.81±2.60 | 4.43±2.46 | <0.001 |
| **Creatinine (μmol/L)** |  | 71.0±27.1 | 56.9±14.8 | <0.001 |
| **Creatinine Clearance Rate** |  | 141.8±53.8 | 176.5±59.4 | <0.001 |
| **Uric Acid (μmol/L)** |  | 418.0±104.5 | 374.6±99.9 | <0.001 |
| Fasting Blood-Glucose (mmol/L) |  | 4.76±1.26 | 4.58±1.16 | 0.067 |
| **Serum Sodium (mmol/L)** |  | 134.8±14.4 | 137.2±2.5 | <0.05 |
| Serum Potassium (mmol/L) |  | 4.17±0.57 | 4.50±6.25 | 0.472 |
| Serum Chloride (mmol/L) |  | 105.62±11.24 | 106.82±6.83 | 0.085 |
| **Serum Calcium (mmol/L)** |  | 1.97±0.19 | 2.06±0.17 | <0.001 |
| **Serum Phosphorus (mmol/L)** |  | 1.37±0.25 | 1.30±0.24 | <0.01 |
| Urine Specific Gravity |  | 1.022±0.011 | 1.071±0.671 | 0.324 |
| **Urine pH** |  | 6.09±0.64 | 6.26±0.68 | <0.01 |
| Urine Leukocytes Count |  | 13.3 (1.0-49.3) | 8.0 (1.0-40.6) | 0.611 |
| **Urine Protein** | negative | 5 (2.7%) | 106 (19.2%) | <0.001 |
|  | (±) | 10 (5.5%) | 54 (9.8%) |  |
|  | (+) | 18 (9.9%) | 106 (19.2%) |  |
|  | (++) | 53 (29.1%) | 124 (22.5%) |  |
|  | (+++) | 72 (39.6%) | 120 (21.8%) |  |
|  | (++++) | 24 (13.2%) | 41 (7.4%) |  |
| Urine Erythrocytes Count |  | 12.2 (1.0-36.3) | 9.5 (1.0-26.1) | 0.199 |
| **Urine Glucose** | negative | 161 (88.5%) | 519 (94.2%) | <0.05 |
|  | (±) | 16 (8.8%) | 14 (2.5%) |  |
|  | (+) | 2 (1.1%) | 10 (1.8%) |  |
|  | (++) | 3 (1.6%) | 5 (0.9%) |  |
|  | (+++) | 0 (0%) | 2 (0.4%) |  |
|  | (++++) | 0 (0%) | 1 (0.2%) |  |
| **Urine Ketone** | negative | 171 (94.0%) | 490 (88.9%) | <0.05 |
|  | (±) | 4 (2.2%) | 20 (3.6%) |  |
|  | (+) | 2 (1.1%) | 6 (1.1%) |  |
|  | (++) | 3 (1.6%) | 20 (3.6%) |  |
|  | (+++) | 2 (1.1%) | 9 (1.6%) |  |
|  | (++++) | 0 (0%) | 6 (1.1%) |  |
| **Urinary Casts** |  | 1.7 (0.8-4.4) | 1.3 (0.1-3.7) | <0.01 |
| **24-hour Urinary Protein (mg)** |  | 3929.2 (1809.0-9380.0) | 1860.0 (366.0-5716.3) | <0.001 |
| **Cholesterol (mmol/L)** |  | 7.08±2.13 | 6.70±2.07 | <0.05 |
| Triglyceride (mmol/L) |  | 4.63±2.64 | 4.25±2.41 | 0.073 |
| ULTRASONIC EXAMINATION |  |  |  |  |
| Amniotic Fluid Index (cm) |  | 6.8±3.8 | 6.7±3.6 | 0.856 |

The variables with bold font indicate there is statistical significance between two groups.
