# Supplementary material for: Comparison of machine learning and logistic regression as predictive models for adverse maternal and neonatal outcomes of preeclampsia: A retrospective study
Source: Front Cardiovasc Med. 2022 Oct 12;9:959649. doi: 10.3389/fcvm.2022.959649 (PMC9596815; doi:10.3389/fcvm.2022.959649)
Supplement: Supplementary Table 4 — Confusing matrixes of predictive models. [file Table_4.DOCX]

Supplementary 4. Confusing matrixes of predictive models developed from training dataset performing imputation.

| **Adverse maternal outcomes** | | True outcome | | **Placental abruption** | | True outcome | |
| --- | --- | --- | --- | --- | --- | --- | --- |
| K-Nearest Neighbor | | Negative(0) | Positive(1) | K-Nearest Neighbor | | Negative(0) | Positive(1) |
| Predicted outcome | Negative(0) | 150 | 19 | Predicted outcome | Negative(0) | 190 | 14 |
|  | Positive(1) | 5 | 46 |  | Positive(1) | 12 | 4 |
| Decision tree classifier | |  |  | Decision tree classifier | |  |  |
| Predicted outcome | Negative(0) | 150 | 10 | Predicted outcome | Negative(0) | 183 | 14 |
|  | Positive(1) | 5 | 55 |  | Positive(1) | 19 | 4 |
| Random forest classifier | |  |  | Random forest classifier | |  |  |
| Predicted outcome | Negative(0) | 154 | 56 | Predicted outcome | Negative(0) | 202 | 18 |
|  | Positive(1) | 1 | 9 |  | Positive(1) | 0 | 0 |
| Support Vector Machine | |  |  | Support Vector Machine | |  |  |
| Predicted outcome | Negative(0) | 143 | 5 | Predicted outcome | Negative(0) | 202 | 17 |
|  | Positive(1) | 12 | 60 |  | Positive(1) | 0 | 1 |
| Multi-Layer Perceptron | |  |  | Multi-Layer Perceptron | |  |  |
| Predicted outcome | Negative(0) | 143 | 5 | Predicted outcome | Negative(0) | 202 | 17 |
|  | Positive(1) | 12 | 60 |  | Positive(1) | 0 | 1 |
| Linear Discriminant Analysis | |  |  | Linear Discriminant Analysis | |  |  |
| Predicted outcome | Negative(0) | 153 | 11 | Predicted outcome | Negative(0) | 196 | 15 |
|  | Positive(1) | 2 | 54 |  | Positive(1) | 6 | 3 |
| Logistic regression | |  |  | Logistic regression | |  |  |
| Predicted outcome | Negative(0) | 153 | 11 | Predicted outcome | Negative(0) | 196 | 15 |
|  | Positive(1) | 2 | 54 |  | Positive(1) | 6 | 3 |
| **Adverse neonatal outcomes** | | True outcome | | **Low birth weight** | | True outcome | |
| K-Nearest Neighbor | | Negative(0) | Positive(1) | K-Nearest Neighbor | | Negative(0) | Positive(1) |
| Predicted outcome | Negative(0) | 78 | 20 | Predicted outcome | Negative(0) | 121 | 32 |
|  | Positive(1) | 13 | 109 |  | Positive(1) | 21 | 46 |
| Decision tree classifier | |  |  | Decision tree classifier | |  |  |
| Predicted outcome | Negative(0) | 80 | 6 | Predicted outcome | Negative(0) | 128 | 23 |
|  | Positive(1) | 11 | 123 |  | Positive(1) | 14 | 55 |
| Random forest classifier | |  |  | Random forest classifier | |  |  |
| Predicted outcome | Negative(0) | 80 | 8 | Predicted outcome | Negative(0) | 133 | 45 |
|  | Positive(1) | 11 | 121 |  | Positive(1) | 9 | 33 |
| Support Vector Machine | |  |  | Support Vector Machine | |  |  |
| Predicted outcome | Negative(0) | 79 | 10 | Predicted outcome | Negative(0) | 129 | 22 |
|  | Positive(1) | 12 | 119 |  | Positive(1) | 13 | 56 |
| Multi-Layer Perceptron | |  |  | Multi-Layer Perceptron | |  |  |
| Predicted outcome | Negative(0) | 79 | 10 | Predicted outcome | Negative(0) | 129 | 22 |
|  | Positive(1) | 12 | 119 |  | Positive(1) | 13 | 56 |
| Linear Discriminant Analysis | |  |  | Linear Discriminant Analysis | |  |  |
| Predicted outcome | Negative(0) | 80 | 12 | Predicted outcome | Negative(0) | 124 | 17 |
|  | Positive(1) | 11 | 117 |  | Positive(1) | 18 | 61 |
| Logistic regression | |  |  | Logistic regression | |  |  |
| Predicted outcome | Negative(0) | 80 | 12 | Predicted outcome | Negative(0) | 124 | 17 |
|  | Positive(1) | 11 | 117 |  | Positive(1) | 18 | 61 |

70% of samples were used as training data and 30% were testing data, therefore, the number of samples in confusion matrixes is 220.
