# Supplementary material for: Comparison of machine learning and logistic regression as predictive models for adverse maternal and neonatal outcomes of preeclampsia: A retrospective study
Source: Front Cardiovasc Med. 2022 Oct 12;9:959649. doi: 10.3389/fcvm.2022.959649 (PMC9596815; doi:10.3389/fcvm.2022.959649)
Supplement: Supplementary Table 5 — Statistical description and test of variables between the placental abruption and control groups. [file Table_5.DOCX]

Supplementary 5. Statistical description and test of variables between the placental abruption group and the control group.

| Variables |  | Study group | Control group | *P* value |
| --- | --- | --- | --- | --- |
| DEMOGRAPHY |  |  |  |  |
| Age (years) |  | 31.9±4.6 | 31.2±5.3 | 0.281 |
| Age over 35 | Yes | 9 (12.7%) | 40 (6.0%) | 0.061 |
| **Gravidity** |  | 2 (1-3) | 2 (1-3) | <0.05 |
| Parity |  | 0 (0-1) | 0 (0-1) | 0.085 |
| COMPLICATIONS |  |  |  |  |
| Chronic Hypertension | Yes | 10 (14.1%) | 87 (13.1%) | 0.824 |
| Diabetes (Pregestational or Gestational) | Yes | 9 (12.7%) | 143 (21.6%) | 0.078 |
| Thyroid Disease | Yes | 3 (4.2%) | 60 (9.1%) | 0.167 |
| IVF-ET | Yes | 1 (1.4%) | 19 (2.9%) | 0.737 |
| Scarred Uterus | Yes | 14 (19.7%) | 83 (12.5%) | 0.090 |
| Twin Pregnancy | Yes | 0 (0%) | 29 (4.4%) | 0.139 |
| **Early-onset type** | Yes | 49 (69.0%) | 227 (34.3%) | <0.001 |
| **Maternal Hypoproteinemia** | Yes | 24 (33.8%) | 132 (19.9%) | <0.01 |
| Thrombocytopenia | Yes | 9 (12.7%) | 50 (7.6%) | 0.132 |
| Impaired Liver Function | Yes | 3 (4.2%) | 34 (5.1%) | 0.962 |
| Cardiovascular disease | Yes | 5 (7.0%) | 21 (3.2%) | 0.181 |
| Renal Insufficiency | Yes | 4 (5.6%) | 21 (3.2%) | 0.458 |
| HELLP Syndrome | Yes | 6 (8.5%) | 35 (5.3%) | 0.406 |
| Postpartum Hemorrhage | Yes | 4 (5.6%) | 13 (2.0%) | 0.124 |
| Eclampsia | Yes | 2 (2.8%) | 14 (2.1%) | 1.000 |
| FEATURE OF DELIVERIES |  |  |  |  |
| **Gestational Age (weeks)** |  | 33.2±3.2 | 35.4±4.3 | <0.001 |
| **Delivery Mode** | vaginal delivery | 0 (0%) | 59 (8.9%) | <0.001 |
|  | forceps delivery | 0 (0%) | 3 (0.5%) |  |
|  | cesarean section | 65 (91.5%) | 535 (80.8%) |  |
|  | 2nd-trimester labor induction | 0 (0%) | 50 (7.6%) |  |
|  | stillbirth delivery | 6 (8.5%) | 15 (2.3%) |  |
| FEATURE OF NEONATES |  |  |  |  |
| Gender of Neonates | Male | 31 (43.7%) | 314 (47.4%) | 0.545 |
| Neonatal Death or Stillbirth | Yes | 6 (8.5%) | 65 (9.8%) | 0.711 |
| **Admitted to NICU** | Yes | 52 (73.2%) | 250 (37.8%) | <0.001 |
| **Low Birth Weight** | Yes | 34 (47.9%) | 219 (33.1%) | <0.05 |
| **Birth Weight of Neonates (g)** |  | 1810.5±743.6 | 2431.8±1039.1 | <0.001 |
| **Apgar Score (1 min)** |  | 8 (3-10) | 9 (8-10) | <0.01 |
| **Apgar Score (5 min)** |  | 10 (8-10) | 10 (10-10) | <0.01 |
| PHYSICAL EXAMINATION |  |  |  |  |
| **Weight (kg)** |  | 76.9±10.1 | 82.0±13.9 | <0.001 |
| Height (cm) |  | 163.9±4.1 | 164.0±4.2 | 0.857 |
| **BMI** |  | 28.6±3.4 | 30.4±4.5 | <0.001 |
| Systolic Pressure (mmHg) |  | 152.3±23.7 | 149.8±23.4 | 0.399 |
| Diastolic Pressure (mmHg) |  | 99.6±18.3 | 96.6±16.5 | 0.153 |
| LABORATORY EXAMINATION |  |  |  |  |
| **Leukocyte ( × 10(9)/L)** |  | 12.74±10.40 | 9.80±3.35 | <0.05 |
| **Neutrophil ( × 10(9)/L)** |  | 62.60 (9.13-78.40) | 14.51 (6.24-71.47) | <0.01 |
| Erythrocyte ( × 10(12)/L) |  | 3.81±0.59 | 4.19±4.73 | 0.495 |
| Hemoglobin (g/L) |  | 119.25±22.79 | 121.11±18.85 | 0.441 |
| Hematokrit (%) |  | 35.45±6.74 | 36.65±5.86 | 0.106 |
| **Platelet ( × 10(9)/L)** |  | 162.16±58.90 | 184.70±69.34 | <0.01 |
| PT (s) |  | 11.10±1.98 | 11.16±5.56 | 0.924 |
| APTT (s) |  | 32.19±13.38 | 30.12±5.03 | 0.198 |
| **Fbg (g/L)** |  | 3.84±1.20 | 4.30±1.54 | <0.05 |
| TT (s) |  | 16.43±2.04 | 16.16±5.42 | 0.670 |
| **ALT (U/L)** |  | 21.0 (15.0-28.0) | 18.0 (13.0-26.0) | <0.05 |
| AST (U/L) |  | 19.0 (11.0-28.0) | 18.0 (12.0-25.5) | 0.841 |
| **Total Protein (g/L)** |  | 52.91±7.28 | 55.55±7.19 | <0.01 |
| **Albumin (g/L)** |  | 28.21±4.21 | 30.20±4.76 | <0.01 |
| Globulin (g/L) |  | 24.80±4.52 | 26.04±19.95 | 0.601 |
| **Urea (mmol/L)** |  | 5.83±2.30 | 4.68±2.59 | <0.001 |
| **Creatinine (μmol/L)** |  | 67.60±20.49 | 59.86±19.69 | <0.01 |
| **Creatinine Clearance Rate** |  | 141.28±44.34 | 169.33±61.83 | <0.001 |
| Uric Acid (μmol/L) |  | 404.74±86.06 | 383.67±105.06 | 0.103 |
| **Fasting Blood-Glucose (mmol/L)** |  | 4.36±0.90 | 4.66±1.22 | <0.05 |
| Serum Sodium (mmol/L) |  | 134.24±16.20 | 136.86±5.88 | 0.180 |
| Serum Potassium (mmol/L) |  | 4.31±0.64 | 4.43±5.71 | 0.856 |
| Serum Chloride (mmol/L) |  | 104.26±17.62 | 106.78±6.34 | 0.234 |
| **Serum Calcium (mmol/L)** |  | 1.93±0.24 | 2.04±0.18 | <0.001 |
| Serum Phosphorus (mmol/L) |  | 1.37±0.19 | 1.32±0.25 | 0.060 |
| Urine Specific Gravity |  | 1.021±0.010 | 1.063±0.613 | 0.572 |
| Urine pH |  | 6.23±0.73 | 6.23±0.67 | 0.978 |
| Urine Leukocytes Count |  | 15.6 (2.0-48.4) | 8.0 (1.0-41.7) | 0.261 |
| **Urine Protein** | negative | 2 (2.8%) | 110 (16.6%) | <0.001 |
|  | (±) | 3 (4.2%) | 60 (9.1%) |  |
|  | (+) | 6 (8.5%) | 120 (18.1%) |  |
|  | (++) | 20 (28.2%) | 158 (23.9%) |  |
|  | (+++) | 34 (47.9%) | 158 (23.9%) |  |
|  | (++++) | 6 (8.5%) | 56 (8.5%) |  |
| Urine Erythrocytes Count |  | 13.3 (1.4-62.8) | 10.6 (1.0-26.1) | 0.053 |
| Urine Glucose | negative | 64 (90.1%) | 618 (93.4%) | 0.394 |
|  | (±) | 7 (9.9%) | 21 (3.2%) |  |
|  | (+) | 0 (0%) | 12 (1.8%) |  |
|  | (++) | 0 (0%) | 8 (1.2%) |  |
|  | (+++) | 0 (0%) | 2 (0.3%) |  |
|  | (++++) | 0 (0%) | 1 (0.2%) |  |
| **Urine Ketone** | negative | 69 (97.2%) | 593 (89.6%) | <0.05 |
|  | (±) | 0 (0%) | 24 (3.6%) |  |
|  | (+) | 1 (1.4%) | 6 (0.9%) |  |
|  | (++) | 1 (1.4%) | 22 (3.3%) |  |
|  | (+++) | 0 (0%) | 11 (1.7%) |  |
|  | (++++) | 0 (0%) | 6 (1.0%) |  |
| Urinary Casts |  | 1.98 (0.70-5.00) | 1.27 (0.13-3.79) | 0.172 |
| **24-hour Urinary Protein (mg)** |  | 6330.0 (2206.9-10370.0) | 2061.5 (495.5-6120.0) | <0.001 |
| Cholesterol (mmol/L) |  | 7.24±2.36 | 6.70±2.03 | 0.069 |
| Triglyceride (mmol/L) |  | 4.52±2.52 | 4.30±2.36 | 0.458 |
| ULTRASONIC EXAMINATION |  |  |  |  |
| Amniotic Fluid Index (cm) |  | 6.5±3.8 | 6.7±3.6 | 0.698 |

The variables with bold font indicate there is statistical significance between two groups.
