# Supplementary material for: Comparison of machine learning and logistic regression as predictive models for adverse maternal and neonatal outcomes of preeclampsia: A retrospective study
Source: Front Cardiovasc Med. 2022 Oct 12;9:959649. doi: 10.3389/fcvm.2022.959649 (PMC9596815; doi:10.3389/fcvm.2022.959649)
Supplement: Supplementary Table 6 — Statistical description and test of variables between the adverse neonatal outcomes group and the control group. [file Table_6.DOCX]

Supplementary 6. Statistical description and test of variables between the adverse neonatal outcomes group and the control group.

| Variables |  | Study group | Control group | *P* value |
| --- | --- | --- | --- | --- |
| DEMOGRAPHY |  |  |  |  |
| Age (years) |  | 31.4±5.6 | 30.9±4.8 | 0.195 |
| Age over 35 | Yes | 26 (6.1%) | 23 (7.4%) | 0.495 |
| **Gravidity** |  | 2 (1-3) | 2 (1-2) | <0.01 |
| **Parity** |  | 0 (0-1) | 0 (0-1) | <0.01 |
| COMPLICATIONS |  |  |  |  |
| Chronic Hypertension | Yes | 62 (14.7%) | 35 (11.3%) | 0.184 |
| Diabetes (Pregestational or Gestational) | Yes | 93 (22.0%) | 59 (19.0%) | 0.330 |
| **Thyroid Disease** | Yes | 48 (11.3%) | 15 (4.8%) | <0.01 |
| IVF-ET | Yes | 15 (3.5%) | 5 (1.6%) | 0.112 |
| Scarred Uterus | Yes | 62 (14.7%) | 35 (11.3%) | 0.184 |
| Twin Pregnancy | Yes | 18 (4.3%) | 11 (3.5%) | 0.628 |
| **Early-onset type** | Yes | 268 (63.4%) | 8 (2.6%) | <0.001 |
| **Maternal Hypoproteinemia** | Yes | 127 (30.0%) | 29 (9.4%) | <0.001 |
| **Thrombocytopenia** | Yes | 53 (12.5%) | 7 (2.3%) | <0.001 |
| **Impaired Liver Function** | Yes | 34 (8.0%) | 4 (1.3%) | <0.001 |
| **Cardiovascular disease** | Yes | 20 (4.7%) | 6 (1.9%) | <0.05 |
| **Renal Insufficiency** | Yes | 24 (5.7%) | 1 (0.3%) | <0.001 |
| **Placental Abruption** | Yes | 61 (14.4%) | 10 (3.2%) | <0.001 |
| **HELLP Syndrome** | Yes | 36 (8.5%) | 5 (1.6%) | <0.001 |
| Postpartum Hemorrhage | Yes | 8 (1.9%) | 9 (2.9%) | 0.369 |
| Eclampsia | Yes | 9 (2.1%) | 7 (2.3%) | 0.905 |
| FEATURE OF DELIVERIES |  |  |  |  |
| **Gestational Age (weeks)** |  | 32.9±4.0 | 38.3±1.9 | <0.001 |
| **Delivery Mode** | vaginal delivery | 14 (3.3%) | 45 (14.5%) | <0.001 |
|  | forceps delivery | 0 (0%) | 3 (1.0%) |  |
|  | cesarean section | 338 (79.9%) | 262 (84.5%) |  |
|  | 2nd-trimester labor induction | 50 (11.8%) | 0 (0%) |  |
|  | stillbirth delivery | 21 (5.0%) | 0 (0%) |  |
| FEATURE OF NEONATES |  |  |  |  |
| Gender of Neonates | Male | 188 (44.4%) | 152 (49.0%) | 0.219 |
| **Birth Weight of Neonates (g)** |  | 1736.3±777.2 | 3247.9±627.4 | <0.001 |
| **Apgar Score (1 min)** |  | 8 (5-10) | 10 (9-10) | <0.001 |
| **Apgar Score (5 min)** |  | 10 (8-10) | 10 (10-10) | <0.001 |
| PHYSICAL EXAMINATION |  |  |  |  |
| **Weight (kg)** |  | 79.6±12.2 | 83.8±15.6 | <0.001 |
| Height (cm) |  | 164.2±3.9 | 163.9±4.8 | 0.264 |
| **BMI** |  | 29.5±3.9 | 31.1±5.1 | <0.001 |
| **Systolic Pressure (mmHg)** |  | 152.8±25.0 | 146.3±20.4 | <0.001 |
| **Diastolic Pressure (mmHg)** |  | 98.4±18.3 | 94.8±14.0 | <0.01 |
| LABORATORY EXAMINATION |  |  |  |  |
| **Leukocyte ( × 10(9)/L)** |  | 10.58±3.76 | 9.48±5.36 | <0.01 |
| **Neutrophil ( × 10(9)/L)** |  | 63.00 (8.44-74.93) | 7.18 (5.52-51.60) | <0.001 |
| Erythrocyte ( × 10(12)/L) |  | 3.93±0.61 | 4.44±6.89 | 0.130 |
| **Hemoglobin (g/L)** |  | 122.5±22.4 | 118.3±15.1 | <0.01 |
| **Hematokrit (%)** |  | 36.89±6.85 | 35.97±4.81 | <0.05 |
| **Platelet ( × 10(9)/L)** |  | 172.24±75.01 | 197.88±55.31 | <0.001 |
| **PT (s)** |  | 10.66±1.45 | 11.83±7.92 | <0.01 |
| APTT (s) |  | 30.44±6.64 | 30.22±5.89 | 0.645 |
| **Fbg (g/L)** |  | 4.11±1.42 | 4.55±1.59 | <0.001 |
| TT (s) |  | 16.30±6.65 | 16.05±1.78 | 0.525 |
| **ALT (U/L)** |  | 21.0 (16.0-32.0) | 14.0 (10.0-20.0) | <0.001 |
| **AST (U/L)** |  | 18.0 (12.0-30.0) | 17.0 (12.0-22.1) | <0.05 |
| **Total Protein (g/L)** |  | 53.13±7.29 | 58.15±6.24 | <0.001 |
| **Albumin (g/L)** |  | 28.62±4.57 | 31.90±4.19 | <0.001 |
| **Globulin (g/L)** |  | 24.56±5.16 | 27.81±28.53 | <0.05 |
| **Urea (mmol/L)** |  | 5.43±3.02 | 3.93±1.38 | <0.001 |
| **Creatinine (μmol/L)** |  | 64.90±22.16 | 54.35±12.29 | <0.001 |
| **Creatinine Clearance Rate** |  | 151.76±54.15 | 185.90±64.35 | <0.001 |
| **Uric Acid (μmol/L)** |  | 407.77±106.88 | 349.90±89.27 | <0.001 |
| Fasting Blood-Glucose (mmol/L) |  | 4.63±1.24 | 4.64±1.16 | 0.909 |
| **Serum Sodium (mmol/L)** |  | 136.17±9.67 | 137.29±2.76 | <0.05 |
| Serum Potassium (mmol/L) |  | 4.44±5.06 | 4.05±0.39 | 0.175 |
| Serum Chloride (mmol/L) |  | 106.88±7.66 | 106.05±8.80 | 0.176 |
| **Serum Calcium (mmol/L)** |  | 2.01±0.20 | 2.07±0.15 | <0.001 |
| **Serum Phosphorus (mmol/L)** |  | 1.36±0.26 | 1.26±0.20 | <0.001 |
| Urine Specific Gravity |  | 1.086±0.765 | 1.021±0.024 | 0.083 |
| Urine pH |  | 6.19±0.66 | 6.21±0.68 | 0.661 |
| **Urine Leukocytes Count** |  | 22.24 (2.10-65.05) | 2.78 (1.00-15.00) | <0.001 |
| **Urine Protein** | negative | 25 (5.9%) | 83 (26.8%) | <0.001 |
|  | (±) | 20 (4.7%) | 43 (13.9%) |  |
|  | (+) | 53 (12.5%) | 72 (23.2%) |  |
|  | (++) | 125 (29.6%) | 57 (18.4%) |  |
|  | (+++) | 152 (35.9%) | 42 (13.5%) |  |
|  | (++++) | 48 (11.3%) | 13 (4.2%) |  |
| **Urine Erythrocytes Count** |  | 17.79 (6.67-38.92) | 1.98 (0.00-11.12) | <0.001 |
| Urine Glucose | negative | 394 (93.1%) | 286 (92.3%) | 0.550 |
|  | (±) | 22 (5.2%) | 6 (1.9%) |  |
|  | (+) | 3 (0.7%) | 9 (2.9%) |  |
|  | (++) | 4 (0.9%) | 6 (1.9%) |  |
|  | (+++) | 0 (0%) | 2 (0.6%) |  |
|  | (++++) | 0 (0%) | 1 (0.3%) |  |
| **Urine Ketone** | negative | 389 (92.0%) | 270 (87.1%) | <0.05 |
|  | (±) | 13 (3.1%) | 12 (3.9%) |  |
|  | (+) | 4 (0.9%) | 4 (1.3%) |  |
|  | (++) | 14 (3.3%) | 12 (3.8%) |  |
|  | (+++) | 1 (0.2%) | 9 (2.9%) |  |
|  | (++++) | 2 (0.5) | 3 (0.9%) |  |
| **Urinary Casts** |  | 1.97 (0.90-5.12) | 0.88 (0.00-2.36) | <0.001 |
| **24-hour Urinary Protein (mg)** |  | 5520.0 (2063.8-9820.0) | 675.6 (236.6-1984.5) | <0.001 |
| **Cholesterol (mmol/L)** |  | 7.12±2.46 | 6.37±1.28 | <0.001 |
| **Triglyceride (mmol/L)** |  | 4.57±2.94 | 3.89±1.32 | <0.001 |
| ULTRASONIC EXAMINATION |  |  |  |  |
| **Amniotic Fluid Index (cm)** |  | 5.66±3.06 | 8.27±3.93 | <0.001 |

The variables with bold font indicate there is statistical significance between two groups.
