# Supplementary material for: Comparison of machine learning and logistic regression as predictive models for adverse maternal and neonatal outcomes of preeclampsia: A retrospective study
Source: Front Cardiovasc Med. 2022 Oct 12;9:959649. doi: 10.3389/fcvm.2022.959649 (PMC9596815; doi:10.3389/fcvm.2022.959649)
Supplement: Supplementary Table 7 — Statistical description and test of variables between the low birth weight group and the control group. [file Table_7.DOCX]

Supplementary 7. Statistical description and test of variables between the low birth weight group and the control group.

| Variables |  | Study group | Control group | *P* value |
| --- | --- | --- | --- | --- |
| DEMOGRAPHY |  |  |  |  |
| Age (years) |  | 30.9±5.4 | 31.4±5.2 | 0.298 |
| Age over 35 | Yes | 13 (5.1%) | 36 (7.5%) | 0.224 |
| Gravidity |  | 2 (1-3) | 2 (1-3) | 0.093 |
| Parity |  | 0 (0-1) | 0 (0-1) | 0.569 |
| COMPLICATIONS |  |  |  |  |
| Chronic Hypertension | Yes | 32 (12.6%) | 65 (13.5%) | 0.734 |
| Diabetes (Pregestational or Gestational) | Yes | 52 (20.6%) | 100 (20.8%) | 0.929 |
| **Thyroid Disease** | Yes | 33 (13.0%) | 30 (6.3%) | <0.01 |
| IVF-ET | Yes | 4 (1.6%) | 16 (3.3%) | 0.166 |
| Scarred Uterus | Yes | 42 (16.6%) | 55 (11.5%) | 0.051 |
| **Twin Pregnancy** | Yes | 2 (0.8%) | 27 (5.6%) | <0.01 |
| **Maternal Hypoproteinemia** | Yes | 79 (31.2%) | 77 (16.0%) | <0.001 |
| Thrombocytopenia | Yes | 27 (10.7%) | 32 (6.7%) | 0.058 |
| Impaired Liver Function | Yes | 16 (6.3%) | 21 (4.4%) | 0.252 |
| Cardiovascular disease | Yes | 11 (4.3%) | 14 (2.9%) | 0.310 |
| Renal Insufficiency | Yes | 16 (6.3%) | 25 (5.2%) | 0.532 |
| **Placental Abruption** | Yes | 34 (13.4%) | 37 (7.7%) | <0.05 |
| HELLP Syndrome | Yes | 9 (3.6%) | 17 (3.5%) | 0.991 |
| Postpartum Hemorrhage | Yes | 4 (1.6%) | 13 (2.7%) | 0.335 |
| Eclampsia | Yes | 5 (2.0%) | 11 (2.3%) | 0.781 |
| FEATURE OF DELIVERIES |  |  |  |  |
| **Gestational Age (weeks)** |  | 34.0 (31.4-36.1) | 37.3 (34.9-39.0) | <0.001 |
| **Delivery Mode** | vaginal delivery | 7 (2.8%) | 52 (10.8%) | <0.001 |
|  | forceps delivery | 0 (0%) | 3 (0.6%) |  |
|  | cesarean section | 222 (87.7%) | 378 (78.8%) |  |
|  | 2nd-trimester labor induction | 20 (7.9%) | 30 (6.3%) |  |
|  | stillbirth delivery | 4 (1.6%) | 17 (3.5%) |  |
| FEATURE OF NEONATES |  |  |  |  |
| **Gender of Neonates** | Male | 102 (40.3%) | 238 (49.6%) | <0.05 |
| Neonatal Death or Stillbirth | Yes | 24 (9.5%) | 47 (9.8%) | 0.894 |
| **Admitted to NICU** | Yes | 179 (70.8%) | 123 (25.6%) | <0.001 |
| **Birth Weight of Neonates (g)** |  | 1547.8±501.1 | 2814.9±974.6 | <0.001 |
| **Apgar Score (1 min)** |  | 9 (7-10) | 9 (9-10) | <0.001 |
| **Apgar Score (5 min)** |  | 10 (9-10) | 10 (10-10) | <0.01 |
| PHYSICAL EXAMINATION |  |  |  |  |
| **Weight (kg)** |  | 78.8±11.5 | 82.9±14.8 | <0.001 |
| Height (cm) |  | 164.1±4.0 | 164.0±4.4 | 0.731 |
| **BMI** |  | 29.1±3.6 | 30.7±4.8 | <0.001 |
| **Systolic Pressure (mmHg)** |  | 152.6±23.5 | 148.7±23.2 | <0.05 |
| Diastolic Pressure (mmHg) |  | 98.2±17.3 | 96.2±16.4 | 0.135 |
| LABORATORY EXAMINATION |  |  |  |  |
| Leukocyte ( × 10(9)/L) |  | 9.70 (7.96-11.53%) | 9.24 (7.48-11.26) | 0.100 |
| **Neutrophil ( × 10(9)/L)** |  | 59.80(7.96-74.67) | 9.28 (5.94-69.74) | <0.001 |
| Erythrocyte ( × 10(12)/L) |  | 3.98±0.62 | 4.24±5.55 | 0.448 |
| **Hemoglobin (g/L)** |  | 125.4±20.8 | 118.4±18.6 | <0.001 |
| **Hematokrit (%)** |  | 37.6±6.8 | 35.9±5.5 | <0.001 |
| Platelet ( × 10(9)/L) |  | 176.7±78.0 | 186.6±62.9 | 0.084 |
| **PT (s)** |  | 10.55±1.56 | 11.47±6.43 | <0.05 |
| APTT (s) |  | 30.41±3.79 | 30.32±7.34 | 0.814 |
| **Fbg (g/L)** |  | 4.05±1.52 | 4.42±1.48 | <0.01 |
| TT (s) |  | 16.48±8.52 | 16.05±1.65 | 0.286 |
| **ALT (U/L)** |  | 22.0 (16.5-33.0) | 17.0 (11.0-23.0) | <0.001 |
| **AST (U/L)** |  | 19.0 (13.0-30.0) | 17.0 (12.0-24.0) | <0.01 |
| **Total Protein (g/L)** |  | 53.27±6.87 | 56.24±7.28 | <0.001 |
| **Albumin (g/L)** |  | 28.79±4.17 | 30.65±4.84 | <0.001 |
| Globulin (g/L) |  | 24.43±5.02 | 26.69±23.19 | 0.126 |
| **Urea (mmol/L)** |  | 5.47±2.14 | 4.44±2.71 | <0.001 |
| **Creatinine (μmol/L)** |  | 64.35±19.99 | 58.41±18.66 | <0.001 |
| **Creatinine Clearance Rate** |  | 150.87±54.68 | 175.04±63.49 | <0.001 |
| **Uric Acid (μmol/L)** |  | 419.24±114.77 | 364.08±91.59 | <0.001 |
| Fasting Blood-Glucose (mmol/L) |  | 4.58±1.14 | 4.67±1.25 | 0.377 |
| Serum Sodium (mmol/L) |  | 136.17±8.79 | 136.91±6.85 | 0.213 |
| Serum Potassium (mmol/L) |  | 4.25±0.50 | 4.28±4.75 | 0.916 |
| Serum Chloride (mmol/L) |  | 107.02±6.83 | 106.26±8.78 | 0.229 |
| **Serum Calcium (mmol/L)** |  | 2.01±0.19 | 2.05±0.18 | <0.01 |
| **Serum Phosphorus (mmol/L)** |  | 1.40±0.27 | 1.28±0.21 | <0.001 |
| Urine Specific Gravity |  | 1.129±0.988 | 1.021±0.020 | 0.083 |
| Urine pH |  | 6.23±0.71 | 6.19±0.64 | 0.516 |
| **Urine Leukocytes Count** |  | 21.68 (2.00-54.49) | 4.65 (1.00-31.14) | <0.001 |
| **Urine Protein** | negative | 20 (7.9%) | 88 (18.3%) | <0.001 |
|  | (±) | 6 (2.4%) | 57 (11.9%) |  |
|  | (+) | 33 (13.0%) | 92 (19.2%) |  |
|  | (++) | 72 (28.5%) | 110 (22.9%) |  |
|  | (+++) | 91 (36.0%) | 103 (21.5%) |  |
|  | (++++) | 31 (12.3%) | 30 (6.3%) |  |
| **Urine Erythrocytes Count** |  | 16.68 (4.78-32.25) | 6.67 (0.45-23.91) | <0.001 |
| Urine Glucose | negative | 233 (92.1%) | 447 (93.1%) | 0.708 |
|  | (±) | 17 (6.7) | 11 (2.3%) |  |
|  | (+) | 1 (0.4%) | 11 (2.3%) |  |
|  | (++) | 2 (0.8%) | 8 (1.7%) |  |
|  | (+++) | 0 (0%) | 2 (0.4%) |  |
|  | (++++) | 0 (0%) | 1 (0.2) |  |
| **Urine Ketone** | negative | 238 (94.1%) | 420 (87.5%) | <0.01 |
|  | (±) | 5 (2.0%) | 20 (4.2%) |  |
|  | (+) | 3 (1.2%) | 5 (1.0%) |  |
|  | (++) | 5 (2.0%) | 22 (4.6%) |  |
|  | (+++) | 0 (0%) | 9 (1.9%) |  |
|  | (++++) | 2 (0.8%) | 4 (0.8%) |  |
| **Urinary Casts** |  | 2.15 (0.88-5.38) | 1.00 (0.00-2.77) | <0.001 |
| **24-hour Urinary Protein (mg)** |  | 5064.7 (1960.2-10940.0) | 1380.0 (350.0-4820.4) | <0.001 |
| **Cholesterol (mmol/L)** |  | 7.37±2.78 | 6.47±1.50 | <0.001 |
| **Triglyceride (mmol/L)** |  | 4.62±3.00 | 4.08±2.01 | <0.05 |
| ULTRASONIC EXAMINATION |  |  |  |  |
| **Amniotic Fluid Index (cm)** |  | 5.7±3.2 | 7.3±3.8 | <0.001 |

The variables with bold font indicate there is statistical significance between two groups.
